# The effect of a Mentor Mothers program on prevention of vertical transmission of HIV outcomes in Zambézia Province, Mozambique: a retrospective interrupted time series analysis

**DOI:** 10.1002/jia2.25952

**Published:** 2022-06-19

**Authors:** James G. Carlucci, Zhihong Yu, Purificación González, Magdalena Bravo, Gustavo Amorim, Cristina das Felicidades Cugara, Helga Guambe, Jaime Mucanhenga, Wilson Silva, José A. Tique, Maria Fernanda Sardella Alvim, Erin Graves, Caroline De Schacht, C. William Wester

**Affiliations:** ^1^ Ryan White Center for Pediatric Infectious Diseases and Global Health, Department of Pediatrics Indiana University School of Medicine Indianapolis Indiana USA; ^2^ Department of Biostatistics Vanderbilt University Medical Center Nashville Tennessee USA; ^3^ Friends in Global Health Quelimane Mozambique; ^4^ Friends in Global Health Maputo Mozambique; ^5^ Ministry of Health Provincial Health Directorate of Zambézia Quelimane Mozambique; ^6^ Ministry of Health National Directorate of Public Health Maputo Mozambique; ^7^ Vanderbilt Institute for Global Health Vanderbilt University Medical Center Nashville Tennessee USA; ^8^ Division of Infectious Diseases, Department of Medicine Vanderbilt University Medical Center Nashville Tennessee USA

**Keywords:** HIV/AIDS, prevention of vertical transmission, maternal–child health, viral suppression, peer support, Mozambique

## Abstract

**Introduction:**

Mentor Mothers (MM) provide peer support to pregnant and postpartum women living with HIV (PPWH) and their infants with perinatal HIV exposure (IPE) throughout the cascade of prevention of vertical transmission (PVT) services. MM were implemented in Zambézia Province, Mozambique starting in August 2017. This evaluation aimed to determine the effect of MM on PVT outcomes.

**Methods:**

A retrospective interrupted time series analysis was done using routinely collected aggregate data from 85 public health facilities providing HIV services in nine districts of Zambézia. All PPWH (and their IPE) who initiated antiretroviral therapy (ART) from August 2016 through April 2019 were included. Outcomes included the proportion per month per district of: PPWH retained in care 12 months after ART initiation, PPWH with viral suppression and IPE with HIV DNA PCR test positivity by 9 months of age. The effect of MM on outcomes was assessed using logistic regression.

**Results:**

The odds of 12‐month retention increased 1.5% per month in the pre‐MM period, compared to a monthly increase of 7.6% with‐MM (35–61% pre‐MM, 56–72% with‐MM; *p* < 0.001). The odds of being virally suppressed decreased by 0.9% per month in the pre‐MM period, compared to a monthly increase of 3.9% with‐MM (49–85% pre‐MM, 59–80% with‐MM; *p* < 0.001). The odds of DNA PCR positivity by 9 months of age decreased 8.9% per month in the pre‐MM period, compared to a monthly decrease of 0.4% with‐MM (0–14% pre‐MM, 4–10% with‐MM; *p* < 0.001). The odds of DNA PCR uptake (the proportion of IPE who received DNA PCR testing) by 9 months of age were significantly higher in the with‐MM period compared to the pre‐MM period (48–100% pre‐MM, 87–100% with‐MM; *p* < 0.001).

**Conclusions:**

MM services were associated with improved retention in PVT services and higher viral suppression rates among PPWH. While there was ongoing but diminishing improvement in DNA PCR positivity rates among IPE following MM implementation, this might be explained by increased uptake of HIV testing among high‐risk IPE who were previously not getting tested. Additional efforts are needed to further optimize PVT outcomes, and MM should be one part of a comprehensive strategy to address this critical need.

## 1 INTRODUCTION

Prevention of vertical transmission of HIV (PVT) services are essential to decreasing new HIV infections among children. The cascade of PVT services includes lifelong antiretroviral therapy (ART) for pregnant and postpartum women living with HIV (PPWH), prophylactic antiretrovirals for infants with perinatal HIV exposure (IPE) and serial HIV testing to ensure timely diagnosis and ART initiation for IPE with confirmed HIV infection. Sustained maternal ART promotes viral suppression, optimizes maternal health outcomes and minimizes the risk of vertical transmission.

Zambézia Province is a rural region of Mozambique that has a population of 5.5 million, an HIV prevalence of 15.1% and a vertical transmission rate of 6–18% [1, 2, 3]. Friends in Global Health (FGH), a subsidiary of Vanderbilt University Medical Center (VUMC), has been providing technical assistance for HIV services, including PVT, in Zambézia since 2006. These services are provided under the auspices of the United States Centers for Disease Control and Prevention (CDC) and the President's Emergency Plan for AIDS Relief.

VUMC/FGH started implementing a Mentor Mothers (MM) program in 2017. The MM program is a peer support service, through which HIV‐affected women assist PPWH and their IPE as they navigate the cascade of PVT services. At least monthly, MM conduct preventative and problem‐focused home visits and/or phone calls to PPWH, beginning at enrolment in antenatal care (ANC). MM conduct these and other activities through linkage with a dedicated health facility (HF), at a ratio of one MM to approximately 20 PPWH. The same MM provides support to the mother–baby dyad during the pregnancy and postpartum periods, until the IPE has been discharged from the Clinic for Children at Risk (CCR) after definitive HIV testing is performed (typically after 18 months of age and cessation of breastfeeding).

This evaluation aimed to assess the impact of MM on retention of PPWH in PVT services, viral suppression among PPWH and HIV DNA PCR test positivity rates (proxy for vertical transmission) among IPE in Zambézia.

## 2 METHODS

### 2.1 Study design and setting

This was a retrospective interrupted time series analysis using routinely collected patient data from VUMC/FGH‐supported HF in Zambézia Province, Mozambique. During the study period (August 2016–April 2019), the province was comprised of 18 districts in which there were 230 public HF, and VUMC/FGH supported 112 of these. Each district‐level health system consists of one large central HF/referral centre and smaller peripheral HF. The VUMC/FGH MM program was implemented at HF providing maternal–child health services (including ANC), and the number of MM affiliated with each HF was proportionate to the volume of PPWH served at each HF (Table S1). Unpublished FGH programmatic data indicate that during the study period, 25–53% of pregnant women living with HIV were not receiving ART at time of presentation to ANC, and ≥97% of these women were started on ART under the prevailing ART initiation policies (initially Option B+, then Test and Start) [1, 2, 3, 4].

### 2.2 Inclusion and exclusion criteria

All PPWH and their IPE were eligible for inclusion if they: (1) enrolled in PVT services at one of 85 VUMC/FGH‐supported HF in nine districts (Table S1); (2) newly initiated ART in ANC during the current pregnancy; and (3) enrolled in care from August 2016 (1‐year pre‐MM implementation in August 2017) to April 2019 (end of evaluation period; however, the MM program continues at all sites).

We excluded 27 HF that: (1) did not support maternal–child health services; (2) were supported by mothers2mothers® (M2M)—a similar but independent mentoring program for PPWH that had been implemented at a small proportion of VUMC/FGH‐supported HF (i.e. excluded to ensure comparability across intervention sites); or (3) were not supported by VUMC/FGH during the pre‐MM period (i.e. those HF in the district of Quelimane). An additional 15 HF were excluded from some analyses because of systematic missingness of outcomes data (i.e. non‐random missingness before or after a certain time point), as specified in Table S1.

### 2.3 Outcomes, definitions and data sources

Our primary outcomes were retention of PPWH in PVT services, viral suppression among PPWH and HIV DNA PCR test positivity rates among IPE. *Retention* among PPWH was defined relative to time from ART initiation in ANC; we determined the proportion of PPWH per month who were still in care at 1, 3, 6 and 12 months after ART initiation (see the Supplementary Methods for individual‐level retention definitions). Each month, the number of PPWH who initiated ART 1, 3, 6 and 12 months prior and the number of PPWH who were still in care were recorded in the Open Medical Record System (OpenMRS)™ for each HF. The retention proportions for each district were calculated using the aggregated district‐level numbers.

*Viral suppression* among PPWH was defined as a viral load (HIV RNA PCR) <1000 copies/ml. For this analysis, we determined the proportion of PPWH per month per district with viral suppression among all available viral load results for PPWH during the period of observation. Each month, the number of PPWH who had viral load testing and the number of PPWH who were virally suppressed were recorded in OpenMRS for each HF. Viral suppression proportions for each district were calculated using the aggregated district‐level numbers.

*HIV DNA PCR test positivity* among IPE was determined for the periods 0–2 months and 0–9 months postpartum. This was defined as the proportion of positive DNA PCR results among all DNA PCR tests performed during the specified period. Each month, the number of DNA PCR tests for IPE within 0–2 months and 0–9 months and the corresponding number of positive DNA PCR results were recorded in District Health Information Software (DHIS) for each HF. The DNA PCR positivity rates for each district were calculated using the aggregated district‐level numbers.

Other outcomes of interest included the uptake of HIV DNA PCR testing, uptake of ANC, institutional delivery and registration of IPE in CCR. *Uptake of HIV DNA PCR testing* among IPE was determined for the periods 0–2 months and 0–9 months postpartum. Each month, the number of DNA PCR tests for IPE within 0–2 months and 0–9 months were recorded in DHIS, but the exact number of IPE eligible for testing was unknown, so the number of pregnant women living with HIV who registered at ANC 6 months earlier was used as a proxy denominator. Descriptions of the methods and results pertaining to the uptake of ANC, institutional delivery and registration of IPE in CCR can be found in the Supplementary Materials (Supplementary Methods, Results and Tables S2–S4).

OpenMRS and DHIS data from 85 HF in nine districts were included. Data were captured from August 2016 through April 2019. For each of the outcomes, we used aggregate data from each HF included in the evaluation. Routinely collected, de‐identified data were extracted from both databases for this retrospective interrupted time series analysis.

### 2.4 Statistical analyses

To account for the phased implementation of MM services at various HFs (Table S1), outcomes were assessed at the HF level, accounting for respective implementation dates and looking at 12 months before and 12 months after MM implementation. Then, the pre/post outcomes were aggregated at the district level for comparison. Specifically, monthly HF‐level data were aggregated to district level by: (1) setting the MM start timepoint for each HF; (2) defining a new variable for each HF representing the MM implementation months (*mm_month* = the calendar year/month – MM start year/month); and (3) aggregating HF data within each district based on *mm_month*.

Since all outcomes varied temporally (i.e. from −12 to +12 *mm_month*) and spatially (i.e. across nine districts), descriptive statistics were calculated within each district for pre‐MM and with‐MM periods separately, as well as for the entire study period. Each descriptive statistic was then compared across all nine districts.

A proxy denominator was used to determine the uptake of HIV DNA PCR testing, which led to 17 (7.6%) of 225 proportions being >1. To address these invalid proportions, three approaches were employed for a sensitivity analysis: (1) cap all proportions >1 at a value of 1; (2) randomly replace each of them with a number between 0.9 and 1; and (3) exclude all invalid proportions from analysis. All three approaches yielded similar results, so only the results from the first approach (i.e. capping at 1) are reported.

For each outcome, we assessed the effect of MM implementation via interrupted time series analysis using monthly district‐level aggregate data. Specifically, an indicator variable named *mm* was defined by assigning “no” for the pre‐MM period and “yes” otherwise, and a multivariable logistic regression model focusing on *mm*, *mm_month* and *district* was built to explore the effect of MM implementation adjusted by district. Sensitivity analyses were performed using logistic regression with a quasibinomial link function to account for potential overdispersion and generalized linear mixed‐effect models to account for clustering (see details in Supplementary Methods), and the results were essentially the same as those from the original fixed effect models (Tables S5 and S6).

Statistical analyses were conducted using R statistical software 3.6.3 [5].

### 2.5 Ethical considerations and assurances

This data use and evaluation plan was approved by the VUMC Institutional Review Board (#201887), the Institutional Research Ethics Committee for Health of Zambézia Province (#16‐CIBS‐Z‐18) and was reviewed in accordance with the CDC human research protection procedures and was determined to be research, but CDC investigators did not interact with human subjects or have access to identifiable data or specimens for research purposes. Individual informed consent was not required for this evaluation since only routinely collected, de‐identified, aggregate data were used, and a waiver of informed consent was approved under the umbrella program evaluation protocol covering this analysis.

## 3 RESULTS

### 3.1 Retention 1 month after ART initiation

**Figure 1:**
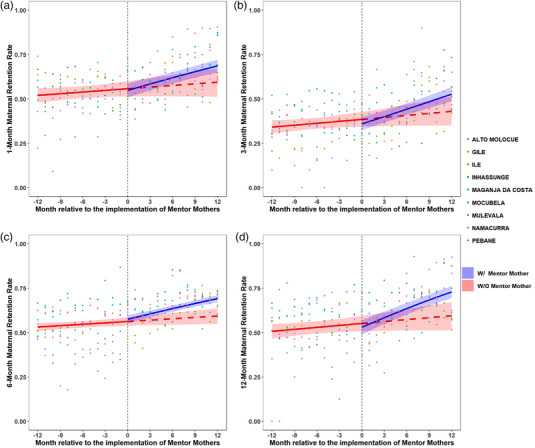
Retention rate for pregnant and postpartum women living with HIV (PPWH). (a) 1‐month retention. (b) 3‐month retention. (c) 6‐month retention. (d) 12‐month retention. The vertical dashed line represents the start of Mentor Mother (MM) implementation, with the 12 months prior to MM implementation to the left and 12 months with MM to the right. The red line represents what happened in the pre‐MM period and projects what would have happened if MM had not been implemented. The blue line represents what was observed after MM implementation. The shaded areas represent the 95% confidence interval of the fitted lines. The dots show the raw data from each district that was used for regression analysis.

In the year pre‐MM, median district‐level 1‐month retention of PPWH was 34–59%. In the year with‐MM, 1‐month retention was 53–71% (Table S7). Province‐wide, the odds of 1‐month retention increased 1.3% per month in the pre‐MM period, compared to an increase of 5% per month with‐MM (*p* = 0.001; Figure 1a [province level] and Figure S1 [district level]).

### 3.2 Retention 3 months after ART initiation

In the year pre‐MM, median district‐level 3‐month retention of PPWH was 15–47%. In the year with‐MM, 3‐month retention was 37–49% (Table S8). Province‐wide, the odds of 3‐month retention increased 1.6% per month in the pre‐MM period, compared to an increase of 6% per month with‐MM (*p* < 0.001; Figure 1b [province level] and Figure S2 [district level]).

### 3.3 Retention 6 months after ART initiation

In the year pre‐MM, median district‐level 6‐month retention of PPWH was 33–67%. In the year with‐MM, 6‐month retention was 56–70% (Table S9). Province‐wide, the odds of 6‐month retention increased 1.1% per month in the pre‐MM period, compared to an increase of 4.3% per month with‐MM (*p* < 0.001; Figure 1c [province level] and Figure S3 [district level]).

### 3.4 Retention 12 months after ART initiation

In the year pre‐MM, median district‐level 12‐month retention of PPWH was 35–61%. In the year with‐MM, 12‐month retention was 56–72% (Table S10). Province‐wide, the odds of 12‐month retention increased 1.5% per month in the pre‐MM period, compared to an increase of 7.6% per month with‐MM (*p* < 0.001; Figure 1d [province level] and Figure S4 [district level]).

### 3.5 Viral suppression

**Figure 2:**
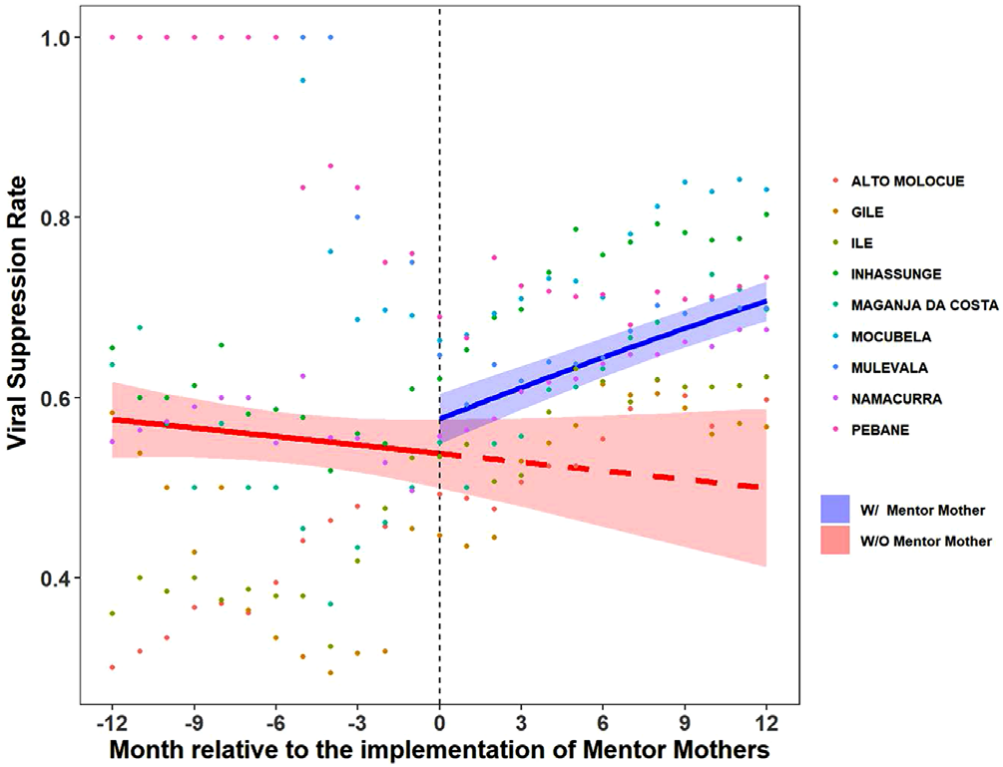
Viral suppression rate for pregnant and postpartum women living with HIV (PPWH). The vertical dashed line represents the start of Mentor Mother (MM) implementation, with the 12 months prior to MM implementation to the left and 12 months with MM to the right. The red line represents what happened in the pre‐MM period and projects what would have happened if MM had not been implemented. The blue line represents what was observed after MM implementation. The shaded areas represent the 95% confidence interval of the fitted lines. The dots show the raw data from each district that was used for regression analysis.

In the year pre‐MM, median district‐level viral suppression among PPWH was 49–85%. In the year with‐MM, viral suppression was 59–80% (Table S11). Province‐wide, the odds of being virally suppressed decreased by 0.9% per month in the pre‐MM period, compared to an increase of 3.9% per month with‐MM (*p* < 0.001; Figure 2 [province level] and Figure S5 [district level]).

### 3.6 Uptake of HIV DNA PCR testing by 2 months of age

**Figure 3:**
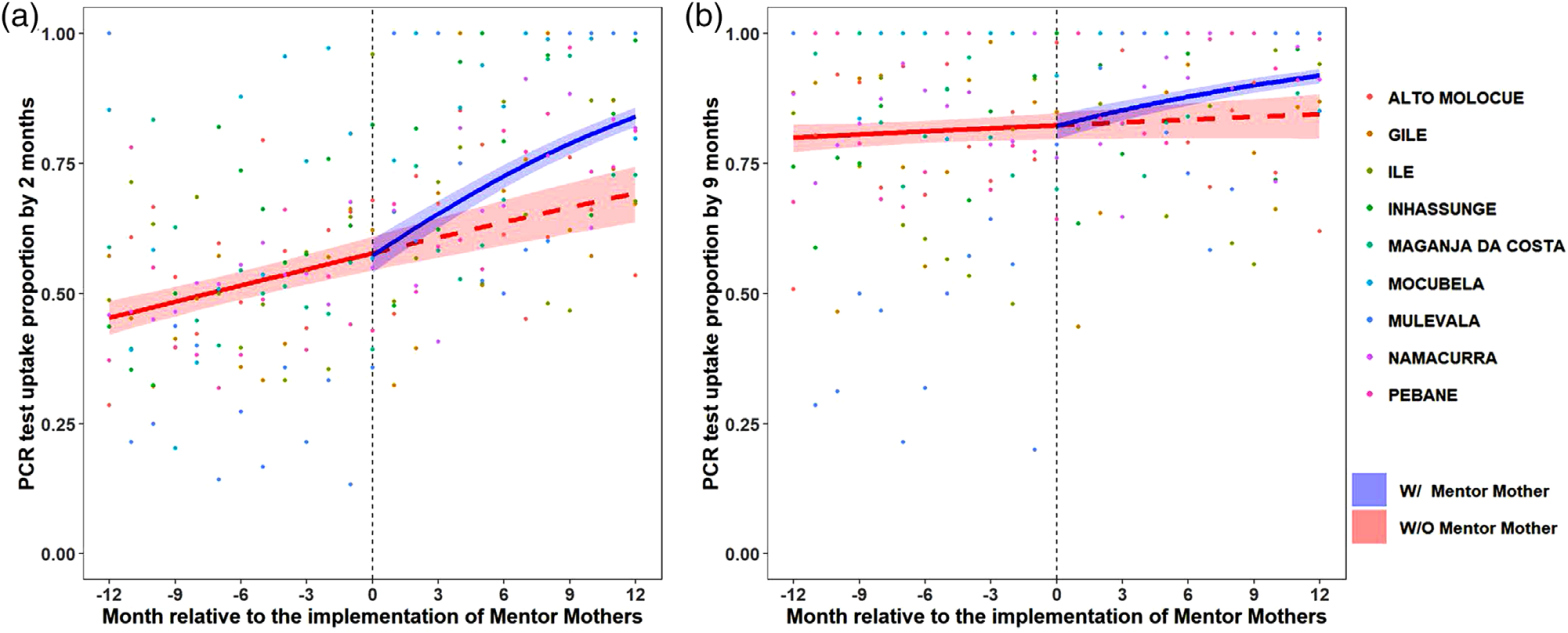
Uptake of HIV DNA PCR testing among infants with perinatal HIV exposure. (a) By age of 2 months. (b) By age of 9 months. The vertical dashed line represents the start of Mentor Mother (MM) implementation, with the 12 months prior to MM implementation to the left and 12 months with MM to the right. The red line represents what happened the pre‐MM and projects what would have happened if MM had not been implemented. The blue line represents what was observed after MM implementation. The shaded areas represent the 95% confidence interval of the fitted lines. The dots show the raw data from each district that was used for regression analysis.

In the year pre‐MM, median district‐level uptake of DNA PCR testing among IPE by 2 months of age was 26–67%. In the year with‐MM, 2‐month DNA PCR uptake was 67–99% (Table S12). Province‐wide, the odds of DNA PCR uptake by 2 months of age increased by 4.4% per month in pre‐MM period, compared to an increase of 12.3% per month with‐MM (*p* < 0.001; Figure 3a [province level] and Figure S6 [district level]).

### 3.7 Uptake of HIV DNA PCR testing by 9 months of age

In the year pre‐MM, median district‐level uptake of DNA PCR testing among IPE by 9 months of age was 48–100%. In the year with‐MM, 9‐month DNA PCR uptake was 87–100% (Table S13). Province‐wide, the odds of DNA PCR uptake by 9 months of age increased by 1.4% per month in pre‐MM period, compared to an increase of 8.1% per month with‐MM (*p* < 0.001; Figure 3b [province level] and Figure S7 [district level]).

### 3.8 HIV DNA PCR test positivity 0–2 months of age

**Figure 4:**
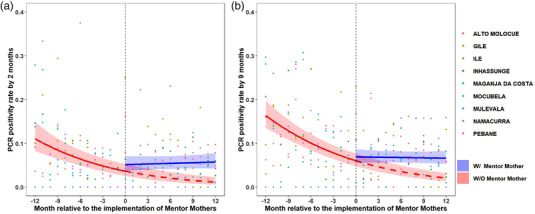
HIV DNA PCR test positivity among tested infants with perinatal HIV exposure. (a) By age of 2 months. (b) By age of 9 months. The vertical dashed line represents the start of Mentor Mother (MM) implementation, with the 12 months prior to MM implementation to the left and 12 months with MM to the right. The red line represents what happened the pre‐MM and projects what would have happened if MM had not been implemented. The blue line represents what was observed after MM implementation. The shaded areas represent the 95% confidence interval of the fitted lines. The dots show the raw data from each district that was used for regression analysis.

In the year pre‐MM, median district‐level DNA PCR positivity rates among IPE tested by 2 months of age were 0–8%. In the year with‐MM, 2‐month DNA PCR positivity was 0–8% (Table S14). The odds of DNA PCR positivity decreased 9.4% per month in the pre‐MM period, compared to an increase of 1% per month with‐MM (*p* < 0.001; Figure 4a [province level] and Figure S8 [district level]).

### 3.9 HIV DNA PCR test positivity 0–9 months of age

In the year pre‐MM, median district‐level DNA PCR positivity rates among IPE tested by 9 months of age were 0–14%. In the year with‐MM, 9‐month DNA PCR positivity was 4–10% (Table S15). The odds of DNA PCR positivity decreased 8.9% per month in the pre‐MM period, compared to a decrease of 0.4% per month with‐MM (*p* < 0.001; Figure 4b [province level] and Figure S9 [district level]).

## 4 DISCUSSION

In this interrupted time series analysis of PVT outcomes, we found that implementation of an MM program was associated with improved retention in PVT services and higher viral suppression rates among PPWH. The proportion of PPWH retained in care was significantly higher in the period with MM implementation compared to the pre‐MM period, and this trend was consistently observed at all time points (1, 3, 6 and 12 months) after ART initiation. This association between MM implementation and retention in care likely contributed to the observed improvements in viral suppression during the period with MM implementation. In other words, sustained engagement in care is the cornerstone for promoting optimal ART adherence and achieving and maintaining viral suppression, and MM have the potential to positively influence these outcomes.

A recent scoping review showed the importance of lay health worker support in the management of PPWH and IPE; however, evidence for lay worker impact on adherence to ART and virologic outcomes was lacking [6]. The results of our study support a positive effect of MM on retention and viral suppression among PPWH. As such, MM may have an important role to play in attaining “the third 95” of the UNAIDS 95‐95‐95 goals (i.e. 95% of those on ART are virally suppressed) [7]. However, even though we saw significant improvements in retention and viral suppression after implementation of MM, retention and viral suppression rates still fell well short of the “third 95” target, perhaps indicative of a ceiling effect and emphasizing the need for a multifaceted strategy that includes but is not limited to MM services. Additional research and investments are needed to understand and realize the full potential of MM and other strategies for optimizing PVT outcomes [8]. There is evidence that peer support through MM approaches is acceptable [9], an important factor for success of these strategies, but other implementation outcomes, including fidelity to the strategy, still need to be explored.

In the pre‐MM period, the proportion of PPWH retained in care was already trending in a positive direction, but the rate of rise significantly increased with MM implementation. It is possible that the pre‐MM improvements in retention were attributable to program maturation or other initiatives aimed at improving retention in care, and that MM implementation led to additional or synergistic gains. In contrast, viral suppression rates were decreasing prior to MM implementation, but increased significantly in the with‐MM period. Decreasing viral suppression rates in the pre‐MM period might have been the result of concurrent gains in retention of a sub‐group of PPWH who previously were neither in care nor virally suppressed. Perhaps during the with‐MM period, PPWH were not only better retained in care but also had better adherence to ART and virologic control because of MM support. Clinical trials have also shown a beneficial impact of peer/mentoring support on maternal PVT outcomes [10, 11, 12]. However, both the Mother Mentor (MoMent) study in Nigeria and the Mother and Infant Retention for Health (MIR4Health) study in Kenya only assessed these outcomes through 6 months postpartum [10, 11], and MIR4Health tested a combination intervention that made it difficult to isolate the relative contribution of MM services to improved outcomes [11]. While our study design is limited in its ability to assess causality, our real‐world assessments of viral suppression and retention in care up to 12 months following ART initiation are strengths.

Despite improvements in viral suppression among PPWH in the with‐MM period, this did not translate into comparable declines in HIV DNA PCR test positivity rates among IPE. In the pre‐MM period, there were steady declines in DNA PCR positivity, with rates approaching 5% or less in most districts; however, in the with‐MM period, there was diminishing improvement in DNA PCR positivity rates among IPE 0–9 months of age, and DNA PCR positivity slightly increased among IPE 0–2 months of age. These observations might be explained by increased uptake of HIV testing in the with‐MM period (i.e. increased testing among IPE at higher risk for vertical transmission, but who were less likely to be tested in the pre‐MM period). In fact, there was a significant increase in the number of IPE enrolled in CCR over time, and the odds of DNA PCR uptake by 2 and 9 months of age were significantly higher in the with‐MM period compared to the pre‐MM period. The MoMent study also demonstrated that MM services were associated with improved rates of IPE presentation for DNA PCR testing by 2 months of age [13]. While the ultimate goal is to eliminate vertical transmission, it should still be viewed as a success if MM are helping high‐risk mother–baby dyads stay in care, access diagnostic testing and get linked to care and treatment when indicated. Furthermore, it may be the case that the retention benefits of MM are more immediate, viral suppression is further down the causal pathway and improvements in vertical transmission are even further downstream; we may need more than 1 year of observation after implementation of MM to realize and measure the full impact of MM on infant outcomes.

While there were clear trends in the aggregate data, there was considerable variability in outcomes between HF and districts. Some of this variability might be attributable to outliers from sites with relatively small patient populations skewing the data, but it is also possible that there was heterogeneity in how MM services were delivered across sites. Other studies support MM as an effective strategy for promoting optimal PVT outcomes, but the extent to which MM impact these outcomes has varied between diverse contexts and depends on the content and implementation of MM strategies [10, 11, 12, 13, 14, 15]. Furthermore, findings from the Mother–Infant Visit Adherence and Treatment Engagement study in Kenya indicate that depression, stigma and intimate partner violence modulate PVT outcomes within the context of MM strategies, indicating a need to develop MM strategies that can respond to the specific needs of HIV‐affected mother–infant dyads [16, 17]. While protocols for training and supervision of MM were consistent across sites included in this evaluation, we were unable to adjust for individual‐level characteristics or fidelity to MM protocols. Ideally, we would have accounted for the extent to which MM supportive and tracing visits were performed, and the content and intensity of the support provided. Implementation research is needed to understand and address the complex needs of HIV‐affected mother–infant dyads, the type of MM interactions that can best serve those needs and organizational factors that will allow for high‐fidelity implementation of MM strategies.

An important limitation of this study was that we were unable to fully assess MM impact on enrolment in ANC and CCR clinics and institutional deliveries (see Supplementary Materials). For each of these outcomes, prospective enrolment and follow‐up of all persons “at‐risk” for the outcome of interest would be necessary. Rather, this was a retrospective study, so the true denominators for each of these outcomes were unknown.

## 5 CONCLUSIONS

We found that MM program implementation was associated with improved retention in PVT services and higher viral suppression rates among PPWH. While there was ongoing but diminishing improvement in HIV DNA PCR test positivity rates among IPE following MM implementation, this might be explained by increased uptake of HIV testing among high‐risk IPE who were previously not getting tested. While it is difficult to infer causality from this study design, and there could have been concurrent but unaccounted for program improvements confounding these results, our findings indicate significant improvements in PVT outcomes associated with the implementation of MM services. Therefore, we recommend strengthening MM services in this context, while concurrently attempting to measure mediators and outcomes that were beyond the scope and resources of this evaluation. Future expansion or adaptation of the MM strategy should be guided by implementation research, which is needed to understand and realize the full potential of MM and other strategies for optimizing PVT outcomes.

## COMPETING INTERESTS

The authors have no competing interests to declare.

## AUTHORS’ CONTRIBUTIONS

JGC, PG, MB, EG, CDS and CWW conceptualized and designed the evaluation. MFSA coordinated data processing and transfer. ZY and GA developed the data analysis plan and analysed the data. ZY produced the tables and figures. JGC, ZY, GA, EG, CDS and CWW synthesized and interpreted the results. PG, MB, CDFC, HG, JM, WS and JAT provided programmatic and contextual insights for understanding the results and guiding the discussion. JGC drafted initial and final drafts of the manuscript, but all authors have read, edited and approved the final manuscript. CDS and CWW provided critical oversight and guidance throughout all stages of the evaluation.

## FUNDING

This evaluation has been supported by the President's Emergency Plan for AIDS Relief (PEPFAR) through the United States Centers for Disease Control and Prevention (CDC) under the terms of cooperative agreements # NU2GGH001943 and # NUGGH002367 (PI/PD: Wester). The findings, conclusions and opinions expressed by authors contributing to this journal are those of the author(s) and do not necessarily represent the official position of the funding agencies or the authors’ affiliated institutions.

## Supporting information


**Figure S1**. One‐month retention rate for pregnant and postpartum women living with HIV (PPWH).
**Figure S2**. Three‐month retention rate for pregnant and postpartum women living with HIV (PPWH).
**Figure S3**. Six‐month retention rate for pregnant and postpartum women living with HIV (PPWH).
**Figure S4**. Twelve‐month retention rate for pregnant and postpartum women living with HIV (PPWH).
**Figure S5**. Viral suppression rate for pregnant and postpartum women living with HIV (PPWH).
**Figure S6**. HIV DNA PCR uptake among infants with perinatal HIV exposure by 2 months of age.
**Figure S7**. HIV DNA PCR uptake among infants with perinatal HIV exposure by 9 months of age.
**Figure S8**. HIV DNA PCR positivity among infants with perinatal HIV exposure tested from 0–2 months of age.
**Figure S9**. HIV DNA PCR positivity among infants with perinatal HIV exposure tested from 0–9 months of age.
**Table S1**. Mentor Mother (MM) program implementation in relation to district/health facility and time period.
**Table S2**. Monthly enrollment of pregnant women living with HIV in antenatal care per district one year before (pre‐MM) and one year after (with‐MM) implementation of MM services.
**Table S3**. Monthly number of pregnant women living with HIV who gave birth at a health facility per district one year before (pre‐MM) and one year after (with‐MM) implementation of MM services.
**Table S4**. Proportion of infants with perinatal HIV exposure who were enrolled in the Clinic for Children at Risk per month per district one year before (pre‐MM) and one year after (with‐MM) implementation of MM services.
**Table S5**. The ratio of residual deviance over the degrees of freedom for all logistic models.
**Table S6**. Model details and comparisons of interested terms in Model #1 to Model #9.
**Table S7**. Proportion of pregnant and postpartum women living with HIV (PPWH) who were retained in care 1‐month after ART initiation per month per district one year before (pre‐MM) and one year after (with‐MM) implementation of MM services.
**Table S8**. Proportion of pregnant and postpartum women living with HIV (PPWH) who were retained in care 3‐months after ART initiation per month per district one year before (pre‐MM) and one year after (with‐MM) implementation of MM services.
**Table S9**. Proportion of pregnant and postpartum women living with HIV (PPWH) who were retained in care 6‐months after ART initiation per month per district one year before (pre‐MM) and one year after (with‐MM) implementation of MM services.
**Table S10**. Proportion of pregnant and postpartum women living with HIV (PPWH) who were retained in care 12‐months after ART initiation per month per district one year before (pre‐MM) and one year after (with‐MM) implementation of MM services.
**Table S11**. Proportion of pregnant and postpartum women living with HIV (PPWH) who were virally suppressed per month per district one year before (pre‐MM) and one year after (with‐MM) implementation of MM services.
**Table S12**. Proportion of infants with perinatal HIV exposure who received HIV DNA PCR testing by 2 months of age per month per district one year before (pre‐MM) and one year after (with‐MM) implementation of MM services.
**Table S13**. Proportion of infants with perinatal HIV exposure who received HIV DNA PCR testing by 9 months of age per month per district one year before (pre‐MM) and one year after (with‐MM) implementation of MM services.
**Table S14**. HIV DNA PCR positivity among infants with perinatal HIV exposure who received DNA PCR testing by 2 months of age per month per district one year before (pre‐MM) and one year after (with‐MM) implementation of MM services.
**Table S15**. HIV DNA PCR positivity among infants with perinatal HIV exposure who received DNA PCR testing by 9 months of age per month per district one year before (pre‐MM) and one year after (with‐MM) implementation of MM services.

## Data Availability

The deidentified datasets used and analysed for the current study are available from the corresponding author upon reasonable request.
