# Supplementary material for: The effect of a Mentor Mothers program on prevention of vertical transmission of HIV outcomes in Zambézia Province, Mozambique: a retrospective interrupted time series analysis
Source: J Int AIDS Soc. 2022 Jun 19;25(6):e25952. doi: 10.1002/jia2.25952 (PMC9207359; doi:10.1002/jia2.25952)
Supplement: Supplementary file 1 — Figure S1. One‐month retention rate for pregnant and postpartum women living with HIV (PPWH). Figure S2. Three‐month retention rate for pregnant and postpartum women living with HIV (PPWH). Figure S3. Six‐month retention rate for pregnant and postpartum women living with HIV (PPWH). Figure S4. Twelve‐month retention rate for pregnant and postpartum women living with HIV (PPWH). Figure S5. Viral suppression rate for pregnant and postpartum women living with HIV (PPWH). Figure S6. HIV DNA PCR uptake among infants with perinatal HIV exposure by 2 months of age. Figure S7. HIV DNA PCR uptake among infants with perinatal HIV exposure by 9 months of age. Figure S8. HIV DNA PCR positivity among infants with perinatal HIV exposure tested from 0–2 months of age. Figure S9. HIV DNA PCR positivity among infants with perinatal HIV exposure tested from 0–9 months of age. Table S1. Mentor Mother (MM) program implementation in relation to district/health facility and time period. Table S2. Monthly enrollment of pregnant women living with HIV in antenatal care per district one year before (pre‐MM) and one year after (with‐MM) implementation of MM services. Table S3. Monthly number of pregnant women living with HIV who gave birth at a health facility per district one year before (pre‐MM) and one year after (with‐MM) implementation of MM services. Table S4. Proportion of infants with perinatal HIV exposure who were enrolled in the Clinic for Children at Risk per month per district one year before (pre‐MM) and one year after (with‐MM) implementation of MM services. Table S5. The ratio of residual deviance over the degrees of freedom for all logistic models. Table S6. Model details and comparisons of interested terms in Model #1 to Model #9. Table S7. Proportion of pregnant and postpartum women living with HIV (PPWH) who were retained in care 1‐month after ART initiation per month per district one year before (pre‐MM) and one year after (with‐MM) implementation of MM services. Table S8 [file JIA2-25-e25952-s001.docx]

**SUPPLEMENTAL MATERIALS**

**Supplemental Methods**

***Individual-level retention definitions***

Individual-level retention definitions were defined as follows:

- 1-month retention: ART pick-up within 1 month (33 days) after ART start
- 3-month retention: ART pick-up within 3 months (99 days) after ART start
- 6-month retention: ART pick-up within 6 months after ART start
- 12-month retention: ART pick-up within 12 months after ART start

***Additional Outcomes, Definitions, and Data Sources***

*Uptake of antenatal care (ANC)* was determined by the absolute number of pregnant women living with HIV who attended their first ANC clinic visit. Ideally, this should be defined as the proportion of pregnant women living with HIV attending their first ANC clinic visit among all eligible to attend their first ANC clinic during the specified time period, but the true denominator could not be determined. Therefore, the absolute number recorded in District Health Information Software (DHIS) monthly for each health facility (HF) were aggregated at the district-level and used for analyses.

*Institutional delivery* was determined by the absolute number of pregnant women living with HIV who registered at maternity wards. Ideally, this should be defined as the proportion of pregnant women living with HIV registered at maternity wards among all pregnant women living with HIV due for delivery during the specified time period, but the true denominator could not be determined. Therefore, the absolute number recorded in DHIS monthly for each HF were aggregated at the district-level and used for analyses.

*Registration of infants with perinatal HIV exposure (IPE) in the Clinic for Children at Risk (CCR)* was determined by the absolute number of IPE registered at CCR. Ideally, this should be defined as the proportion of IPE registered at CCR among all IPE eligible to register at CCR during the specified time period, but the true denominator could not be determined. Therefore, the absolute number recorded in DHIS monthly for each HF were aggregated at the district-level and used for analyses.

For these outcomes in the format of absolute number, a linear regression model was built to assess the effect of implementing Mentor Mothers (MM).

***Interrupted Time Series Analysis***

*Original fixed-effect model using binomial link function:*

For each of the nine outcomes, we assessed the effect of Mentor Mothers (MM) implementation via interrupted time series analysis (ITS) using monthly district-level aggregate data. Specifically, an indicator variable named *mm* was defined by assigning the values “no” for the pre-MM period and “yes” otherwise; an MM implementation time variable in month named *mm_month* was calculated by “*mm_month* = the calendar year/month - MM start year/month”. A multivariable logistic regression model focusing on *mm* and *mm_month* was built to explore the effect of MM implementation adjusted by *District*. An interaction term between *mm_month* and *mm* was included in the initial model, and it was retained if it was statistically significant. Otherwise, a new model without the interaction term was built to assess the effect of MM implementation. The general equation for this model is shown below:

$$logit\left\{ Prob\left( Y=1 \right) \right\}= \beta_{0}+\sum_{i=1}^{8} \beta_{i} I\left( District={distric}_{i} \right)+\beta_{9}mm\_month+\beta_{10}mm+\beta_{11}mm\_month*mm$$

where *Y* is the outcome of interest (e.g., 1-, 3- , 6-, or 12-month retention, viral suppression, etc.); *District* is a categorical variable with “Alto Molocue” as reference and the other 8 districts correspond to 8 indicator variables within the summation notation.

When interpreting the model, we focused on the last three coefficients (i.e. $\beta_{9}, \beta_{10}, and \beta_{11}$) and converted them to odds ratio scale by exponentiating each coefficient. Model 1 with 1-month retention outcome (details shown in Table S6) is used as an example to demonstrate how to interpret these coefficients. $\beta_{9}$ represents the slope of the time variable before the MM program was implemented; ${exp(\beta}_{9})=1.013$ in model 1 means that, before MM implementation, the odds of being retained at 1-month increased by about 1.3% (i.e. 1.013 – 1 = 0.013) at each month, but this change was not statistically significant at the 5% level (the 95% CI contains the value 1). The parameter $\beta_{10}$ represents the level change right after the MM program started, meaning an instantaneous impact of the MM program. The value ${exp(\beta}_{10})=0.964$ in model 1 means that the odds of being retained at 1-month decreased by about 3.6% (i.e. 0.964 – 1 = -0.036) right after the MM program started, but this change was not statistically significant at the 5% level. $\beta_{11}$ measures the slope change after the MM implementation, compared to the counterfactual setting of no MM program; ${exp(\beta}_{11})=1.037$ in model 1 means that, the monthly change for the odds of being retained for 1-month after MM program is 1.037 times of the monthly change before MM program. Finally, for the coefficient of each district indicator variable (i.e. $\beta_{1}-\beta_{8}$), it represents the intercept difference versus the reference district of “Alto Molocue”; ${exp(\beta}_{1})=0.869$ in model 1 means that, when all other variables are held constant, the odds of being retained at 1-month in district “Gile” is about 87% of that in district “Alto Molocue”.

*Sensitivity analysis accounting for potential overdispersion:*

The ratio of residual deviance over the degrees of freedom for all nine ITS models indicate potential overdispersion especially for two PCR testing uptake outcomes (Table S5), which may lead to inaccurate confidence interval for the estimated coefficients. To account for overdispersion, the logit link function in the main analyses was changed to a quasibinomial logit link, which has an extra parameter to estimate the variance. Results are displayed in Table S6.

*Sensitivity analysis accounting for clustering:*

The main analyses are fixed-effect models focusing on the effect of MM program but adjusted for different districts. As another sensitivity analyses, the generalized linear mixed-effect models (GLMM) were also built for comparison. District were treated as a cluster variable and both intercept and *mm* (i.e. the intervention indicator) variable were set as random effects. Results are displayed in Table S6.

**Supplemental Results**

*Uptake of Antenatal Care*

As shown in **Table S2**, the median number of pregnant women living with HIV enrolled in ANC per month ranged from 15 to 171 in the year before MM implementation. In the year during MM implementation, median monthly ANC enrollment ranged from 15 to 188. While there was significant variability in the absolute number of pregnant women living with HIV enrolled in ANC across districts (p<0.001), there was not a statistically significant difference in ANC enrollment numbers before and after MM implementation (p=0.38). Furthermore, the absolute number of pregnant women living with HIV who were eligible for ANC enrollment is unknown, so we were unable to determine if the proportion of eligible pregnant women living with HIV enrolled in ANC changed over time or with respect to implementation of MM services.

*Institutional Delivery*

As shown in **Table S3**, the median number of pregnant women living with HIV who gave birth at a HF per month ranged from 2 to 116 in the year before MM implementation. In the year during MM implementation, median monthly institutional deliveries ranged from 7 to 127. There was significant variability in the absolute number of institutional deliveries across districts (p<0.001). There was also a significant increase in the absolute number of institutional deliveries over time (13.2 per year; p<0.001), but in the period with-MM there were 9 fewer institutional deliveries than would be expected based on data from the pre-MM period (p=0.001). That said, the absolute number of pregnant women living with HIV who should have given birth at a HF is unknown, so we were unable to determine if the proportion of institutional deliveries as a function of all possible deliveries changed over time or with respect to implementation of MM services.

*Clinic for Children at Risk Registration*

As shown in **Table S4**, the median number of IPE enrolled in CCR per month ranged from 8 to 116 in the year before MM implementation. In the year during MM implementation median monthly CCR enrollment ranged from 19 to 165. There was significant variability in the absolute number of IPE enrolled in CCR across districts (p<0.001). There was also a significant increase in the absolute number of IPE enrolled in CCR over time (20.2 per year; p<0.001); however, there was not a statistically significant change in CCR enrollment during the period with-MM compared to the pre-MM period (p=0.67). That said, the absolute number of IPE who should have been enrolled in CCR is unknown, so we were unable to determine if the proportion of IPE enrolled in CCR as a function of all possible CCR enrollments changed over time or with respect to implementation of MM services.

**Supplemental Tables**

**Table S1.** Mentor Mother (MM) program implementation in relation to district/health facility and time period.

| **District**  Health Facility | **Implementation period for MM program** | **Number of MM per health facility** |
| --- | --- | --- |
| **Alto Molòcué** | | |
| CS B. Gruveta ^b^ | Dec 2017 – Feb 2018 | 4 |
| CS Caiaia | Dec 2017 – Feb 2018 | 2 |
| CS Chapala | Dec 2017 – Feb 2018 | 4 |
| CS Cololo | Dec 2017 – Feb 2018 | 1 |
| CS Ecole | Dec 2017 – Feb 2018 | 1 |
| CS Malua | Dec 2017 – Feb 2018 | 2 |
| CS Moiua | Dec 2017 – Feb 2018 | 2 |
| CS Mutala | Dec 2017 – Feb 2018 | 3 |
| CS Nacuacua | Dec 2017 – Feb 2018 | 2 |
| CS Nauela | Dec 2017 – Feb 2018 | 4 |
| CS Nimala | Dec 2017 – Feb 2018 | 2 |
| CS Nivava | Dec 2017 – Feb 2018 | 2 |
| CS Novanana | Dec 2017 – Feb 2018 | 2 |
| HR Alto Molòcué | Nov 2017 – Jan 2018 | 29 |
| **Gilé** | | |
| CS Alto Ligonha | Nov 2017 – Jan 2018 | 3 |
| CS Intxotxa ^a^ | Mar – May 2018 | 2 |
| CS Kayane | Nov 2017 – Jan 2018 | 6 |
| CS Mamala | Nov 2017 – Jan 2018 | 5 |
| CS Moneia | Nov 2017 – Jan 2018 | 6 |
| CS Muiane | Nov 2017 – Jan 2018 | 8 |
| CS Namuaca | Nov 2017 – Jan 2018 | 2 |
| CS Pury | Nov 2017 – Jan 2018 | 3 |
| CS Uape | Nov 2017 – Jan 2018 | 3 |
| HD Gilé | Dec 2017 – Feb 2018 | 13 |
| **Ile** | | |
| CS Curruane ^a^ | Dec 2017 – Feb 2018 | 1 |
| CS Ile | Dec 2017 – Feb 2018 | 15 |
| CS Massira ^a^ | Dec 2017 – Feb 2018 | 1 |
| CS Mucuaba ^a^ | Dec 2017 – Feb 2018 | 3 |
| CS Mugulama | Dec 2017 – Feb 2018 | 8 |
| CS Mulequela | Dec 2017 – Feb 2018 | 4 |
| CS Namanda ^a^ | Dec 2017 – Feb 2018 | 6 |
| CS Niboia | Dec 2017 – Feb 2018 | 2 |
| CS Phalane ^a^ | Dec 2017 – Feb 2018 | 2 |
| CS Socone | Dec 2017 – Feb 2018 | 3 |
| CS Ualasse ^a^ | Dec 2017 – Feb 2018 | 1 |
| **Inhassunge** | | |
| CS Bingagira | Dec 2017 – Feb 2018 | 7 |
| CS Cherimane | Dec 2017 – Feb 2018 | 6 |
| CS Gonhane | Dec 2017 – Feb 2018 | 11 |
| CS Ilova | Dec 2017 – Feb 2018 | 3 |
| CS Inhassunge | Dec 2017 – Feb 2018 | 16 |
| CS Olinda | Dec 2017 – Feb 2018 | 3 |
| CS Palane-Mucula | Dec 2017 – Feb 2018 | 9 |
| **Maganja da Costa** | | |
| CS Alto Mutola | Aug – Oct 2017 | 6 |
| CS Cabuir | Aug – Oct 2017 | 6 |
| CS Cariua | Aug – Oct 2017 | 4 |
| CS Mabala | Aug – Oct 2017 | 8 |
| CS Maganja da Costa | Aug – Oct 2017 | 36 |
| CS Mapira | Aug – Oct 2017 | 2 |
| CS Moneia ^a^ | Aug – Oct 2017 | 3 |
| CS Muzo | Aug – Oct 2017 | 2 |
| CS Nante | Aug – Oct 2017 | 12 |
| **Mocubela** | | |
| CS Gurai | Aug – Oct 2017 | 8 |
| CS Ilha Idugo ^a^ | Sep – Nov 2018 | 6 |
| CS Maneia | Aug – Oct 2017 | 4 |
| CS Mocubela | Aug – Oct 2017 | 11 |
| CS Naico | Aug – Oct 2017 | 8 |
| CS Tapata | Aug – Oct 2017 | 17 |
| **Mulevala** | | |
| CS Chiraco | Feb – Apr 2018 | 4 |
| CS Jajo ^a^ | Feb – Apr 2018 | 3 |
| CS Marropino ^a^ | Apr – Jun 2018 | 2 |
| CS Morrua ^a^ | Apr – Jun 2018 | 1 |
| CS Mulevala | Feb – Apr 2018 | 5 |
| CS Tebo ^a^ | Apr – Jun 2018 | 3 |
| **Namacurra** | | |
| CS Furquia | Oct – Dec 2017 | 19 |
| CS Macuse | Oct – Dec 2017 | 13 |
| CS Malei | Jan – Mar 2018 | 7 |
| CS Mbaua | Oct – Dec 2017 | 13 |
| CS Mixixine | Oct – Dec 2017 | 14 |
| CS Muceliua | Oct – Dec 2017 | 7 |
| CS Muebele | Oct – Dec 2017 | 12 |
| CS Mugubia | Oct – Dec 2017 | 5 |
| CS Mutange ^a^ | Feb – Apr 2018 | 3 |
| CS Namacurra | Oct – Dec 2017 | 24 |
| **Pebane** | | |
| CS 7 Abril | Aug – Oct 2017 | 14 |
| CS Alto Maganha | Aug – Oct 2017 | 8 |
| CS Impaca | Aug – Oct 2017 | 4 |
| CS Magiga | Aug – Oct 2017 | 13 |
| CS Malema | Aug – Oct 2017 | 5 |
| CS Mihecue | Aug – Oct 2017 | 2 |
| CS Mulela | Aug – Oct 2017 | 3 |
| CS Muligode | Aug – Oct 2017 | 4 |
| CS Naburi | Aug – Oct 2017 | 7 |
| CS Pebane | Aug – Oct 2017 | 16 |
| CS Pele-Pele | Aug – Oct 2017 | 6 |
| CS Tomea | Aug – Oct 2017 | 2 |

* This table does not include districts/health facilities that: did not support maternal-child health services, those supported by “Mothers 2 Mothers” (M2M), those who implemented MM services after December 2018, or those in Quelimane District for which we lacked pre-MM data (see exclusion criteria).

^a^ Fourteen health facilities were excluded due to systematic missingness when analyzing outcomes from District Health Information Software (DHIS; i.e., all outcomes except for maternal retention and viral suppression).

^b^ One additional health facility was excluded due to systematic missingness when analyzing the outcome of institutional delivery.

**Table S2.** Monthly enrollment of pregnant women living with HIV in antenatal care per district one year before (pre-MM) and one year after (with-MM) implementation of MM services.

| **District** | **Period** | **Min** | **Q1** | **Median** | **Q3** | **Max** | **Mean** | **SD** |
| --- | --- | --- | --- | --- | --- | --- | --- | --- |
| ALTO MOLÒCUÉ | Pre-MM | 50 | 55 | 57 | 66 | 70 | 59 | 7 |
|  | With-MM | 54 | 62 | 63 | 71 | 74 | 64 | 6 |
|  | Entire period of evaluation | 50 | 56 | 62 | 67 | 74 | 62 | 7 |
| GILÉ | Pre-MM | 43 | 60 | 66 | 67 | 81 | 63 | 10 |
|  | With-MM | 55 | 65 | 67 | 78 | 92 | 71 | 11 |
|  | Entire period of evaluation | 43 | 61 | 66 | 74 | 92 | 67 | 11 |
| ILE | Pre-MM | 25 | 34 | 38 | 45 | 54 | 39 | 8 |
|  | With-MM | 31 | 37 | 39 | 45 | 52 | 41 | 6 |
|  | Entire period of evaluation | 25 | 34 | 39 | 45 | 54 | 40 | 7 |
| INHASSUNGE | Pre-MM | 49 | 56 | 62 | 73 | 84 | 64 | 11 |
|  | With-MM | 65 | 68 | 77 | 84 | 103 | 79 | 12 |
|  | Entire period of evaluation | 49 | 63 | 71 | 80 | 103 | 72 | 14 |
| MAGANJA DA COSTA | Pre-MM | 76 | 93 | 102 | 111 | 140 | 105 | 17 |
|  | With-MM | 81 | 96 | 100 | 113 | 121 | 103 | 11 |
|  | Entire period of evaluation | 76 | 94 | 102 | 113 | 140 | 104 | 14 |
| MOCUBELA | Pre-MM | 33 | 66 | 70 | 83 | 111 | 73 | 19 |
|  | With-MM | 70 | 72 | 82 | 87 | 114 | 83 | 12 |
|  | Entire period of evaluation | 33 | 70 | 80 | 87 | 114 | 78 | 17 |
| MULEVALA | Pre-MM | 8 | 14 | 15 | 16 | 22 | 15 | 4 |
|  | With-MM | 8 | 14 | 15 | 18 | 26 | 16 | 5 |
|  | Entire period of evaluation | 8 | 14 | 15 | 18 | 26 | 15 | 4 |
| NAMACURRA | Pre-MM | 129 | 150 | 171 | 180 | 201 | 166 | 22 |
|  | With-MM | 148 | 172 | 188 | 204 | 214 | 185 | 23 |
|  | Entire period of evaluation | 129 | 159 | 174 | 195 | 214 | 176 | 24 |
| PEBANE | Pre-MM | 121 | 137 | 145 | 158 | 193 | 148 | 20 |
|  | With-MM | 139 | 154 | 167 | 176 | 190 | 165 | 15 |
|  | Entire period of evaluation | 121 | 143 | 155 | 171 | 193 | 157 | 19 |

**Table S3.** Monthly number of pregnant women living with HIV who gave birth at a health facility per district one year before (pre-MM) and one year after (with-MM) implementation of MM services.

| **District** | **Period** | **Min** | **Q1** | **Median** | **Q3** | **Max** | **Mean** | **SD** |
| --- | --- | --- | --- | --- | --- | --- | --- | --- |
| ALTO MOLÒCUÉ | Pre-MM | 22 | 28 | 31 | 38 | 44 | 33 | 7 |
|  | With-MM | 15 | 30 | 33 | 37 | 47 | 33 | 7 |
|  | Entire period of evaluation | 15 | 29 | 31 | 37 | 47 | 33 | 7 |
| GILÉ | Pre-MM | 24 | 36 | 38 | 43 | 50 | 38 | 8 |
|  | With-MM | 27 | 36 | 40 | 45 | 66 | 43 | 12 |
|  | Entire period of evaluation | 24 | 36 | 39 | 44 | 66 | 41 | 10 |
| ILE | Pre-MM | 12 | 14 | 16 | 19 | 26 | 17 | 4 |
|  | With-MM | 8 | 13 | 16 | 18 | 24 | 16 | 4 |
|  | Entire period of evaluation | 8 | 13 | 16 | 19 | 26 | 16 | 4 |
| INHASSUNGE | Pre-MM | 34 | 40 | 52 | 58 | 66 | 50 | 11 |
|  | With-MM | 37 | 52 | 56 | 75 | 80 | 60 | 15 |
|  | Entire period of evaluation | 34 | 45 | 53 | 66 | 80 | 55 | 14 |
| MAGANJA DA COSTA | Pre-MM | 36 | 46 | 58 | 59 | 64 | 53 | 10 |
|  | With-MM | 38 | 50 | 53 | 57 | 75 | 54 | 9 |
|  | Entire period of evaluation | 36 | 49 | 56 | 59 | 75 | 54 | 9 |
| MOCUBELA | Pre-MM | 22 | 30 | 32 | 36 | 50 | 34 | 8 |
|  | With-MM | 28 | 30 | 35 | 42 | 46 | 36 | 7 |
|  | Entire period of evaluation | 22 | 30 | 33 | 40 | 50 | 35 | 7 |
| MULEVALA | Pre-MM | 2 | 2 | 2 | 4 | 8 | 4 | 2 |
|  | With-MM | 1 | 6 | 7 | 7 | 10 | 6 | 3 |
|  | Entire period of evaluation | 1 | 2 | 5 | 7 | 10 | 5 | 3 |
| NAMACURRA | Pre-MM | 88 | 92 | 116 | 122 | 130 | 110 | 16 |
|  | With-MM | 101 | 115 | 127 | 140 | 168 | 128 | 19 |
|  | Entire period of evaluation | 88 | 109 | 118 | 130 | 168 | 119 | 19 |
| PEBANE | Pre-MM | 69 | 92 | 97 | 106 | 118 | 98 | 14 |
|  | With-MM | 87 | 101 | 105 | 116 | 131 | 107 | 13 |
|  | Entire period of evaluation | 69 | 96 | 104 | 115 | 131 | 103 | 14 |

**Table S4.** Proportion of infants with perinatal HIV exposure who were enrolled in the Clinic for Children at Risk per month per district one year before (pre-MM) and one year after (with-MM) implementation of MM services.

| **District** | **Period** | **Min** | **Q1** | **Median** | **Q3** | **Max** | **Mean** | **SD** |
| --- | --- | --- | --- | --- | --- | --- | --- | --- |
| ALTO MOLÒCUÉ | Pre-MM | 24 | 32 | 40 | 48 | 61 | 40 | 11 |
|  | With-MM | 41 | 51 | 57 | 63 | 75 | 57 | 9 |
|  | Entire period of evaluation | 24 | 40 | 50 | 61 | 75 | 49 | 13 |
| GILÉ | Pre-MM | 20 | 35 | 44 | 59 | 75 | 46 | 16 |
|  | With-MM | 40 | 53 | 62 | 75 | 149 | 69 | 27 |
|  | Entire period of evaluation | 20 | 43 | 55 | 62 | 149 | 58 | 25 |
| ILE | Pre-MM | 21 | 26 | 29 | 32 | 55 | 32 | 10 |
|  | With-MM | 27 | 33 | 43 | 60 | 64 | 45 | 14 |
|  | Entire period of evaluation | 21 | 28 | 33 | 48 | 64 | 39 | 14 |
| INHASSUNGE | Pre-MM | 30 | 43 | 60 | 67 | 70 | 55 | 14 |
|  | With-MM | 41 | 70 | 75 | 89 | 102 | 74 | 18 |
|  | Entire period of evaluation | 30 | 52 | 67 | 75 | 102 | 65 | 18 |
| MAGANJA DA COSTA | Pre-MM | 56 | 74 | 81 | 88 | 117 | 82 | 17 |
|  | With-MM | 67 | 90 | 104 | 107 | 138 | 100 | 18 |
|  | Entire period of evaluation | 56 | 76 | 90 | 107 | 138 | 91 | 20 |
| MOCUBELA | Pre-MM | 53 | 62 | 67 | 91 | 121 | 77 | 22 |
|  | With-MM | 84 | 102 | 104 | 111 | 124 | 106 | 10 |
|  | Entire period of evaluation | 53 | 68 | 98 | 109 | 124 | 92 | 22 |
| MULEVALA | Pre-MM | 3 | 6 | 8 | 8 | 13 | 7 | 3 |
|  | With-MM | 7 | 14 | 19 | 21 | 24 | 18 | 5 |
|  | Entire period of evaluation | 3 | 7 | 11 | 19 | 24 | 13 | 7 |
| NAMACURRA | Pre-MM | 99 | 112 | 116 | 145 | 163 | 126 | 22 |
|  | With-MM | 128 | 150 | 165 | 174 | 196 | 163 | 21 |
|  | Entire period of evaluation | 99 | 118 | 150 | 165 | 196 | 145 | 28 |
| PEBANE | Pre-MM | 87 | 103 | 113 | 120 | 141 | 113 | 15 |
|  | With-MM | 112 | 131 | 158 | 169 | 180 | 152 | 23 |
|  | Entire period of evaluation | 87 | 112 | 127 | 158 | 180 | 133 | 28 |

**Table S5.** The ratio of residual deviance over the degrees of freedom for all logistic models.

| **Model** | **Outcome** | **Ratio** |
| --- | --- | --- |
| 1 | 1-month retention | 1.3 |
| 2 | 3-month retention | 1.7 |
| 3 | 6-month retention | 2.5 |
| 4 | 12-month retention | 2.1 |
| 5 | Viral suppression | 1.4 |
| **6** | **PCR testing uptake (0-2 months)** | **8.8** |
| **7** | **PCR testing uptake (0-9 months)** | **12.9** |
| 8 | PCR positivity (0-2 months) | 1.9 |
| 9 | PCR positivity (0-9 months) | 2.9 |

**Note:** The results for Models 6 and 7 are bolded to highlight potential overdispersion. However, considering both PCR testing uptake outcomes were calculated using a proxy denominator (as described in Outcomes, Definitions, and Data Sources section of the Methods), this approximation might have introduced some artificial errors and contributed to the overdispersion.

**Table S6.** Model details and comparisons of interested terms in Model #1 to Model #9.

| **Term** | **Original fixed-effect model (binomial link function)** | | | **Fixed-effect model**  **(quasibinomial link function)** | | | **Mixed effect model (GLMM)**  **(district as cluster variable)** | | |
| --- | --- | --- | --- | --- | --- | --- | --- | --- | --- |
|  | **OR** | **95% CI** | **P** | **OR** | **95% CI** | **P** | **OR** | **95% CI** | **P** |
| ***Model #1: 1-month retention*** | | | | | | | | | |
| **mm_month** | 1.013 | 0.996 – 1.029 | 0.132 | 1.013 | 0.994 – 1.032 | 0.188 | 1.013 | 0.996 – 1.030 | 0.130 |
| **mm** | 0.964 | 0.821 – 1.131 | 0.649 | 0.964 | 0.802 – 1.158 | 0.690 | 0.992 | 0.816 – 1.205 | 0.934 |
| **mm_month*mm** | 1.037 | 1.014 – 1.061 | **0.001** | 1.037 | 1.011 – 1.065 | **0.005** | 1.037 | 1.014 – 1.061 | **0.001** |
| ***Model #2: 3-month retention*** | | | | | | | | | |
| **mm_month** | 1.016 | 0.999 – 1.033 | 0.063 | 1.016 | 0.995 – 1.037 | 0.144 | 1.016 | 0.999 – 1.033 | 0.058 |
| **mm** | 0.899 | 0.767 – 1.054 | 0.191 | 0.899 | 0.734 – 1.101 | 0.303 | 0.935 | 0.749 – 1.167 | 0.553 |
| **mm_month*mm** | 1.043 | 1.020 – 1.066 | **<0.001** | 1.043 | 1.014 – 1.072 | **0.004** | 1.042 | 1.020 – 1.065 | **<0.001** |
| ***Model #3: 6-month retention*** | | | | | | | | | |
| **mm_month** | 1.011 | 1.002 – 1.020 | **0.022** | 1.011 | 0.996 – 1.025 | 0.142 | 1.011 | 1.002 – 1.020 | **0.020** |
| **mm** | 1.056 | 0.966 – 1.154 | 0.232 | 1.056 | 0.918 – 1.214 | 0.443 | 1.186 | 0.883 – 1.594 | 0.258 |
| **mm_month*mm** | 1.032 | 1.020 – 1.045 | **<0.001** | 1.032 | 1.013 – 1.053 | **0.001** | 1.030 | 1.017 – 1.043 | **<0.001** |
| ***Model #4: 12-month retention*** | | | | | | | | | |
| **mm_month** | 1.015 | 0.998 – 1.032 | 0.078 | 1.015 | 0.991 – 1.040 | 0.217 | 1.015 | 0.998 – 1.032 | 0.087 |
| **mm** | 0.918 | 0.785 – 1.074 | 0.287 | 0.918 | 0.733 – 1.150 | 0.455 | 0.934 | 0.721 – 1.208 | 0.601 |
| **mm_month*mm** | 1.060 | 1.036 – 1.084 | **<0.001** | 1.060 | 1.026 – 1.095 | **<0.001** | 1.059 | 1.035 – 1.083 | **<0.001** |
| ***Model #5: Viral suppression*** | | | | | | | | | |
| **mm_month** | 0.987 | 0.967 – 1.007 | 0.199 | 0.987 | 0.968 – 1.007 | 0.188 | 0.974 | 0.954 – 0.995 | **0.015** |
| **mm** | 1.176 | 1.014 – 1.363 | **0.032** | 1.176 | 1.018 – 1.359 | **0.028** | 1.023 | 0.672 – 1.558 | 0.915 |
| **mm_month*mm** | 1.065 | 1.041 – 1.088 | **<0.001** | 1.065 | 1.042 – 1.088 | **<0.001** | 1.079 | 1.054 – 1.104 | **<0.001** |
| ***Model #6: PCR testing uptake (0-2 months)*** | | | | | | | | | |
| **mm_month** | 1.044 | 1.031 – 1.057 | **<0.001** | 1.044 | 1.007 – 1.081 | **0.018** | 1.043 | 1.030 – 1.056 | **<0.001** |
| **mm** | 0.981 | 0.870 – 1.106 | 0.751 | 0.981 | 0.699 – 1.375 | 0.910 | 1.084 | 0.792 – 1.484 | 0.615 |
| **mm_month*mm** | 1.076 | 1.057 – 1.095 | **<0.001** | 1.076 | 1.024 – 1.130 | **0.004** | 1.078 | 1.059 – 1.097 | **<0.001** |
| ***Model #7: PCR testing uptake (0-9 months)*** | | | | | | | | | |
| **mm_month** | 1.014 | 0.997 – 1.030 | 0.109 | 1.014 | 0.957 – 1.074 | 0.645 | 1.012 | 0.996 – 1.029 | 0.147 |
| **mm** | 0.995 | 0.845 – 1.170 | 0.948 | 0.995 | 0.563 – 1.755 | 0.985 | 1.175 | 0.757 – 1.823 | 0.473 |
| **mm_month*mm** | 1.066 | 1.041 – 1.091 | **<0.001** | 1.066 | 0.982 – 1.157 | 0.128 | 1.065 | 1.041 – 1.091 | **<0.001** |
| ***Model #8: PCR positivity (0-2 months)*** | | | | | | | | | |
| **mm_month** | 0.906 | 0.875 – 0.938 | **<0.001** | 0.906 | 0.865 – 0.949 | **<0.001** | 0.905 | 0.875 – 0.937 | **<0.001** |
| **mm** | 1.428 | 1.011 – 2.015 | **0.043** | 1.428 | 0.899 – 2.266 | 0.130 | 1.385 | 0.927 – 2.070 | 0.112 |
| **mm_month*mm** | 1.115 | 1.066 – 1.165 | **<0.001** | 1.115 | 1.050 – 1.183 | **<0.001** | 1.115 | 1.067 – 1.165 | **<0.001** |
| ***Model #9: PCR positivity (0-9 months)*** | | | | | | | | | |
| **mm_month** | 0.911 | 0.892 – 0.931 | **<0.001** | 0.911 | 0.880 – 0.944 | **<0.001** | 0.911 | 0.891 – 0.930 | **<0.001** |
| **mm** | 1.153 | 0.919 – 1.446 | 0.218 | 1.153 | 0.791 – 1.680 | 0.457 | 1.115 | 0.856 – 1.452 | 0.421 |
| **mm_month*mm** | 1.093 | 1.061 – 1.126 | **<0.001** | 1.093 | 1.040 – 1.148 | **<0.001** | 1.093 | 1.061 – 1.126 | **<0.001** |

**Abbreviations:** CI, confidence interval; GLMM, generalized linear mixed-effect models; OR, odds ratio; P, p-value.

**Note:** Values that are statistically significant at the p<0.05 level are highlighted in bold. The original model corresponds to the approach used in the manuscript: fixed-effect model adjusting for districts but ignoring overdispersion. The fixed effect model with quasibinomial link function was used to account for overdispersion. The GLMM corresponds to the mixed-effect model that uses districts as cluster variable and treats intercept and mm indicator variable as random effect.

**Table S7.** Proportion of pregnant and postpartum women living with HIV (PPWH) who were retained in care 1-month after ART initiation per month per district one year before (pre-MM) and one year after (with-MM) implementation of MM services.

| **District** | **Period** | **Min** | **Q1** | **Median** | **Q3** | **Max** | **Mean** | **SD** |
| --- | --- | --- | --- | --- | --- | --- | --- | --- |
| ALTO MOLÒCUÉ | Pre-MM | 0.482 | 0.557 | 0.586 | 0.644 | 0.741 | 0.595 | 0.071 |
|  | With-MM | 0.444 | 0.542 | 0.706 | 0.870 | 0.905 | 0.711 | 0.165 |
|  | Entire period of evaluation | 0.444 | 0.542 | 0.640 | 0.733 | 0.905 | 0.655 | 0.139 |
| GILÉ | Pre-MM | 0.393 | 0.470 | 0.568 | 0.626 | 0.636 | 0.543 | 0.094 |
|  | With-MM | 0.387 | 0.647 | 0.706 | 0.762 | 0.900 | 0.680 | 0.144 |
|  | Entire period of evaluation | 0.387 | 0.500 | 0.629 | 0.706 | 0.900 | 0.614 | 0.139 |
| ILE | Pre-MM | 0.417 | 0.518 | 0.555 | 0.628 | 0.684 | 0.559 | 0.080 |
|  | With-MM | 0.454 | 0.483 | 0.556 | 0.611 | 0.741 | 0.564 | 0.085 |
|  | Entire period of evaluation | 0.417 | 0.515 | 0.556 | 0.615 | 0.741 | 0.562 | 0.081 |
| INHASSUNGE | Pre-MM | 0.429 | 0.464 | 0.555 | 0.616 | 0.676 | 0.550 | 0.090 |
|  | With-MM | 0.528 | 0.583 | 0.618 | 0.684 | 0.857 | 0.654 | 0.102 |
|  | Entire period of evaluation | 0.429 | 0.548 | 0.593 | 0.658 | 0.857 | 0.604 | 0.108 |
| MAGANJA DA COSTA | Pre-MM | 0.453 | 0.482 | 0.531 | 0.550 | 0.617 | 0.525 | 0.054 |
|  | With-MM | 0.298 | 0.500 | 0.526 | 0.627 | 0.696 | 0.546 | 0.112 |
|  | Entire period of evaluation | 0.298 | 0.493 | 0.526 | 0.604 | 0.696 | 0.536 | 0.088 |
| MOCUBELA | Pre-MM | 0.415 | 0.476 | 0.544 | 0.597 | 0.704 | 0.536 | 0.086 |
|  | With-MM | 0.500 | 0.553 | 0.659 | 0.688 | 0.875 | 0.641 | 0.105 |
|  | Entire period of evaluation | 0.415 | 0.500 | 0.594 | 0.675 | 0.875 | 0.591 | 0.109 |
| MULEVALA | Pre-MM | 0.091 | 0.282 | 0.339 | 0.500 | 0.571 | 0.370 | 0.150 |
|  | With-MM | 0.438 | 0.600 | 0.640 | 0.750 | 0.875 | 0.664 | 0.134 |
|  | Entire period of evaluation | 0.091 | 0.385 | 0.538 | 0.640 | 0.875 | 0.523 | 0.204 |
| NAMACURRA | Pre-MM | 0.420 | 0.467 | 0.500 | 0.514 | 0.681 | 0.503 | 0.070 |
|  | With-MM | 0.538 | 0.569 | 0.611 | 0.628 | 0.667 | 0.602 | 0.043 |
|  | Entire period of evaluation | 0.420 | 0.500 | 0.552 | 0.614 | 0.681 | 0.554 | 0.076 |
| PEBANE | Pre-MM | 0.451 | 0.492 | 0.540 | 0.563 | 0.628 | 0.531 | 0.056 |
|  | With-MM | 0.500 | 0.564 | 0.582 | 0.623 | 0.773 | 0.596 | 0.068 |
|  | Entire period of evaluation | 0.451 | 0.520 | 0.564 | 0.598 | 0.773 | 0.564 | 0.070 |

**Table S8.** Proportion of pregnant and postpartum women living with HIV (PPWH) who were retained in care 3-months after ART initiation per month per district one year before (pre-MM) and one year after (with-MM) implementation of MM services.

| **District** | **Period** | **Min** | **Q1** | **Median** | **Q3** | **Max** | **Mean** | **SD** |
| --- | --- | --- | --- | --- | --- | --- | --- | --- |
| ALTO MOLÒCUÉ | Pre-MM | 0.162 | 0.308 | 0.333 | 0.349 | 0.390 | 0.316 | 0.064 |
|  | With-MM | 0.167 | 0.333 | 0.435 | 0.615 | 0.900 | 0.474 | 0.199 |
|  | Entire period of evaluation | 0.162 | 0.310 | 0.345 | 0.435 | 0.900 | 0.398 | 0.168 |
| GILÉ | Pre-MM | 0.214 | 0.270 | 0.341 | 0.396 | 0.442 | 0.333 | 0.083 |
|  | With-MM | 0.250 | 0.300 | 0.419 | 0.581 | 0.710 | 0.451 | 0.170 |
|  | Entire period of evaluation | 0.214 | 0.286 | 0.368 | 0.442 | 0.710 | 0.394 | 0.146 |
| ILE | Pre-MM | 0.167 | 0.251 | 0.368 | 0.395 | 0.486 | 0.339 | 0.104 |
|  | With-MM | 0.182 | 0.304 | 0.368 | 0.404 | 0.600 | 0.381 | 0.121 |
|  | Entire period of evaluation | 0.167 | 0.290 | 0.368 | 0.404 | 0.600 | 0.361 | 0.113 |
| INHASSUNGE | Pre-MM | 0.269 | 0.396 | 0.466 | 0.536 | 0.556 | 0.445 | 0.100 |
|  | With-MM | 0.233 | 0.406 | 0.480 | 0.568 | 0.775 | 0.479 | 0.148 |
|  | Entire period of evaluation | 0.233 | 0.406 | 0.467 | 0.546 | 0.775 | 0.462 | 0.125 |
| MAGANJA DA COSTA | Pre-MM | 0.269 | 0.309 | 0.333 | 0.352 | 0.540 | 0.343 | 0.069 |
|  | With-MM | 0.254 | 0.357 | 0.422 | 0.444 | 0.475 | 0.399 | 0.072 |
|  | Entire period of evaluation | 0.254 | 0.309 | 0.357 | 0.429 | 0.540 | 0.372 | 0.075 |
| MOCUBELA | Pre-MM | 0.293 | 0.390 | 0.432 | 0.499 | 0.529 | 0.433 | 0.076 |
|  | With-MM | 0.256 | 0.378 | 0.490 | 0.562 | 0.733 | 0.477 | 0.143 |
|  | Entire period of evaluation | 0.256 | 0.378 | 0.444 | 0.529 | 0.733 | 0.456 | 0.115 |
| MULEVALA | Pre-MM | 0.000 | 0.100 | 0.154 | 0.308 | 0.429 | 0.185 | 0.139 |
|  | With-MM | 0.217 | 0.364 | 0.476 | 0.636 | 0.667 | 0.489 | 0.152 |
|  | Entire period of evaluation | 0.000 | 0.154 | 0.357 | 0.476 | 0.667 | 0.343 | 0.211 |
| NAMACURRA | Pre-MM | 0.208 | 0.328 | 0.348 | 0.364 | 0.465 | 0.348 | 0.063 |
|  | With-MM | 0.369 | 0.391 | 0.429 | 0.472 | 0.663 | 0.444 | 0.079 |
|  | Entire period of evaluation | 0.208 | 0.353 | 0.381 | 0.444 | 0.663 | 0.398 | 0.086 |
| PEBANE | Pre-MM | 0.278 | 0.341 | 0.391 | 0.430 | 0.486 | 0.383 | 0.065 |
|  | With-MM | 0.369 | 0.418 | 0.468 | 0.485 | 0.630 | 0.461 | 0.070 |
|  | Entire period of evaluation | 0.278 | 0.370 | 0.427 | 0.474 | 0.630 | 0.424 | 0.077 |

**Table S9.** Proportion of pregnant and postpartum women living with HIV (PPWH) who were retained in care 6-months after ART initiation per month per district one year before (pre-MM) and one year after (with-MM) implementation of MM services.

| **District** | **Period** | **Min** | **Q1** | **Median** | **Q3** | **Max** | **Mean** | **SD** |
| --- | --- | --- | --- | --- | --- | --- | --- | --- |
| ALTO MOLÒCUÉ | Pre-MM | 0.370 | 0.438 | 0.467 | 0.487 | 0.564 | 0.467 | 0.049 |
|  | With-MM | 0.580 | 0.595 | 0.658 | 0.720 | 0.765 | 0.663 | 0.064 |
|  | Entire period of evaluation | 0.370 | 0.473 | 0.580 | 0.658 | 0.765 | 0.569 | 0.114 |
| GILÉ | Pre-MM | 0.374 | 0.497 | 0.546 | 0.608 | 0.625 | 0.538 | 0.079 |
|  | With-MM | 0.412 | 0.495 | 0.606 | 0.644 | 0.719 | 0.582 | 0.100 |
|  | Entire period of evaluation | 0.374 | 0.495 | 0.547 | 0.615 | 0.719 | 0.561 | 0.092 |
| ILE | Pre-MM | 0.349 | 0.377 | 0.406 | 0.506 | 0.514 | 0.430 | 0.066 |
|  | With-MM | 0.443 | 0.521 | 0.561 | 0.615 | 0.657 | 0.564 | 0.065 |
|  | Entire period of evaluation | 0.349 | 0.408 | 0.508 | 0.561 | 0.657 | 0.500 | 0.094 |
| INHASSUNGE | Pre-MM | 0.622 | 0.653 | 0.674 | 0.692 | 0.794 | 0.680 | 0.044 |
|  | With-MM | 0.612 | 0.674 | 0.696 | 0.730 | 0.853 | 0.710 | 0.074 |
|  | Entire period of evaluation | 0.612 | 0.654 | 0.686 | 0.719 | 0.853 | 0.696 | 0.062 |
| MAGANJA DA COSTA | Pre-MM | 0.449 | 0.504 | 0.528 | 0.567 | 0.608 | 0.530 | 0.048 |
|  | With-MM | 0.536 | 0.568 | 0.632 | 0.682 | 0.735 | 0.628 | 0.064 |
|  | Entire period of evaluation | 0.449 | 0.529 | 0.568 | 0.632 | 0.735 | 0.581 | 0.075 |
| MOCUBELA | Pre-MM | 0.475 | 0.617 | 0.645 | 0.663 | 0.868 | 0.649 | 0.089 |
|  | With-MM | 0.599 | 0.628 | 0.664 | 0.700 | 0.768 | 0.667 | 0.053 |
|  | Entire period of evaluation | 0.475 | 0.617 | 0.652 | 0.685 | 0.868 | 0.658 | 0.071 |
| MULEVALA | Pre-MM | 0.178 | 0.272 | 0.330 | 0.390 | 0.480 | 0.328 | 0.090 |
|  | With-MM | 0.588 | 0.629 | 0.704 | 0.742 | 0.853 | 0.699 | 0.083 |
|  | Entire period of evaluation | 0.178 | 0.333 | 0.588 | 0.704 | 0.853 | 0.521 | 0.207 |
| NAMACURRA | Pre-MM | 0.486 | 0.528 | 0.552 | 0.569 | 0.639 | 0.552 | 0.043 |
|  | With-MM | 0.560 | 0.582 | 0.639 | 0.694 | 0.710 | 0.635 | 0.055 |
|  | Entire period of evaluation | 0.486 | 0.557 | 0.582 | 0.639 | 0.710 | 0.596 | 0.065 |
| PEBANE | Pre-MM | 0.550 | 0.593 | 0.616 | 0.627 | 0.653 | 0.609 | 0.033 |
|  | With-MM | 0.615 | 0.658 | 0.678 | 0.691 | 0.713 | 0.673 | 0.026 |
|  | Entire period of evaluation | 0.550 | 0.615 | 0.651 | 0.678 | 0.713 | 0.642 | 0.043 |

**Table S10.** Proportion of pregnant and postpartum women living with HIV (PPWH) who were retained in care 12-months after ART initiation per month per district one year before (pre-MM) and one year after (with-MM) implementation of MM services.

| **District** | **Period** | **Min** | **Q1** | **Median** | **Q3** | **Max** | **Mean** | **SD** |
| --- | --- | --- | --- | --- | --- | --- | --- | --- |
| ALTO MOLÒCUÉ | Pre-MM | 0.306 | 0.422 | 0.475 | 0.549 | 0.731 | 0.490 | 0.108 |
|  | With-MM | 0.436 | 0.591 | 0.688 | 0.706 | 0.833 | 0.662 | 0.104 |
|  | Entire period of evaluation | 0.306 | 0.474 | 0.579 | 0.700 | 0.833 | 0.579 | 0.136 |
| GILÉ | Pre-MM | 0.486 | 0.519 | 0.562 | 0.596 | 0.742 | 0.572 | 0.073 |
|  | With-MM | 0.444 | 0.537 | 0.561 | 0.629 | 0.676 | 0.577 | 0.069 |
|  | Entire period of evaluation | 0.444 | 0.520 | 0.561 | 0.629 | 0.742 | 0.575 | 0.070 |
| ILE | Pre-MM | 0.353 | 0.455 | 0.510 | 0.561 | 0.667 | 0.501 | 0.088 |
|  | With-MM | 0.314 | 0.500 | 0.613 | 0.741 | 0.929 | 0.618 | 0.175 |
|  | Entire period of evaluation | 0.314 | 0.471 | 0.560 | 0.650 | 0.929 | 0.562 | 0.150 |
| INHASSUNGE | Pre-MM | 0.433 | 0.523 | 0.594 | 0.649 | 0.769 | 0.585 | 0.097 |
|  | With-MM | 0.682 | 0.720 | 0.724 | 0.778 | 0.895 | 0.752 | 0.067 |
|  | Entire period of evaluation | 0.433 | 0.600 | 0.697 | 0.725 | 0.895 | 0.672 | 0.117 |
| MAGANJA DA COSTA | Pre-MM | 0.466 | 0.524 | 0.606 | 0.627 | 0.780 | 0.596 | 0.089 |
|  | With-MM | 0.454 | 0.561 | 0.583 | 0.648 | 0.700 | 0.590 | 0.070 |
|  | Entire period of evaluation | 0.454 | 0.536 | 0.600 | 0.638 | 0.780 | 0.593 | 0.078 |
| MOCUBELA | Pre-MM | 0.413 | 0.568 | 0.600 | 0.695 | 0.730 | 0.605 | 0.094 |
|  | With-MM | 0.514 | 0.595 | 0.619 | 0.722 | 0.767 | 0.636 | 0.080 |
|  | Entire period of evaluation | 0.413 | 0.585 | 0.614 | 0.696 | 0.767 | 0.621 | 0.086 |
| MULEVALA | Pre-MM | 0.000 | 0.210 | 0.345 | 0.511 | 0.667 | 0.340 | 0.230 |
|  | With-MM | 0.273 | 0.467 | 0.600 | 0.667 | 0.889 | 0.572 | 0.172 |
|  | Entire period of evaluation | 0.000 | 0.333 | 0.500 | 0.625 | 0.889 | 0.461 | 0.230 |
| NAMACURRA | Pre-MM | 0.352 | 0.460 | 0.484 | 0.551 | 0.726 | 0.502 | 0.097 |
|  | With-MM | 0.446 | 0.590 | 0.678 | 0.855 | 0.925 | 0.695 | 0.166 |
|  | Entire period of evaluation | 0.352 | 0.474 | 0.568 | 0.726 | 0.925 | 0.602 | 0.167 |
| PEBANE | Pre-MM | 0.464 | 0.487 | 0.539 | 0.636 | 0.684 | 0.557 | 0.082 |
|  | With-MM | 0.532 | 0.562 | 0.660 | 0.696 | 0.724 | 0.634 | 0.071 |
|  | Entire period of evaluation | 0.464 | 0.532 | 0.597 | 0.676 | 0.724 | 0.597 | 0.084 |

**Table S11.** Proportion of pregnant and postpartum women living with HIV (PPWH) who were virally suppressed per month per district one year before (pre-MM) and one year after (with-MM) implementation of MM services.

| **District** | **Period** | **Min** | **Q1** | **Median** | **Q3** | **Max** | **Mean** | **SD** |
| --- | --- | --- | --- | --- | --- | --- | --- | --- |
| ALTO MOLÒCUÉ | Pre-MM | 0.476 | 0.504 | 0.518 | 0.541 | 0.571 | 0.519 | 0.027 |
|  | With-MM | 0.584 | 0.636 | 0.676 | 0.693 | 0.694 | 0.659 | 0.042 |
|  | Entire period of evaluation | 0.476 | 0.519 | 0.584 | 0.676 | 0.694 | 0.592 | 0.079 |
| GILE | Pre-MM | 0.453 | 0.511 | 0.537 | 0.570 | 0.603 | 0.536 | 0.048 |
|  | With-MM | 0.561 | 0.574 | 0.589 | 0.595 | 0.604 | 0.585 | 0.014 |
|  | Entire period of evaluation | 0.453 | 0.542 | 0.574 | 0.591 | 0.604 | 0.561 | 0.042 |
| ILE | Pre-MM | 0.350 | 0.417 | 0.490 | 0.527 | 0.570 | 0.475 | 0.073 |
|  | With-MM | 0.564 | 0.583 | 0.621 | 0.635 | 0.647 | 0.611 | 0.029 |
|  | Entire period of evaluation | 0.350 | 0.506 | 0.568 | 0.621 | 0.647 | 0.546 | 0.088 |
| INHASSUNGE | Pre-MM | 0.598 | 0.613 | 0.628 | 0.635 | 0.667 | 0.628 | 0.021 |
|  | With-MM | 0.651 | 0.686 | 0.717 | 0.727 | 0.733 | 0.704 | 0.030 |
|  | Entire period of evaluation | 0.598 | 0.628 | 0.655 | 0.717 | 0.733 | 0.668 | 0.046 |
| MAGANJA DA COSTA | Pre-MM | 0.453 | 0.526 | 0.605 | 0.641 | 0.708 | 0.586 | 0.084 |
|  | With-MM | 0.525 | 0.593 | 0.649 | 0.704 | 0.714 | 0.642 | 0.066 |
|  | Entire period of evaluation | 0.453 | 0.554 | 0.635 | 0.686 | 0.714 | 0.615 | 0.079 |
| MOCUBELA | Pre-MM | 0.777 | 0.789 | 0.846 | 0.857 | 0.909 | 0.832 | 0.042 |
|  | With-MM | 0.770 | 0.800 | 0.804 | 0.835 | 0.844 | 0.811 | 0.024 |
|  | Entire period of evaluation | 0.770 | 0.790 | 0.806 | 0.846 | 0.909 | 0.821 | 0.035 |
| MULEVALA | Pre-MM | 0.600 | 0.714 | 0.714 | 1.000 | 1.000 | 0.805 | 0.149 |
|  | With-MM | 0.667 | 0.706 | 0.717 | 0.739 | 0.757 | 0.718 | 0.028 |
|  | Entire period of evaluation | 0.600 | 0.707 | 0.714 | 0.751 | 1.000 | 0.760 | 0.112 |
| NAMACURRA | Pre-MM | 0.510 | 0.535 | 0.543 | 0.566 | 0.572 | 0.546 | 0.019 |
|  | With-MM | 0.583 | 0.612 | 0.649 | 0.699 | 0.711 | 0.649 | 0.049 |
|  | Entire period of evaluation | 0.510 | 0.546 | 0.583 | 0.649 | 0.711 | 0.600 | 0.064 |
| PEBANE | Pre-MM | 0.718 | 0.769 | 0.849 | 0.855 | 0.857 | 0.815 | 0.052 |
|  | With-MM | 0.710 | 0.738 | 0.746 | 0.774 | 0.789 | 0.753 | 0.024 |
|  | Entire period of evaluation | 0.710 | 0.744 | 0.774 | 0.848 | 0.857 | 0.783 | 0.050 |

**Table S12.** Proportion of infants with perinatal HIV exposure who received HIV DNA PCR testing by 2 months of age per month per district one year before (pre-MM) and one year after (with-MM) implementation of MM services.

| **District** | **Period** | **Min** | **Q1** | **Median** | **Q3** | **Max** | **Mean** | **SD** |
| --- | --- | --- | --- | --- | --- | --- | --- | --- |
| ALTO MOLÒCUÉ | Pre-MM | 0.286 | 0.470 | 0.589 | 0.630 | 0.794 | 0.557 | 0.135 |
|  | With-MM | 0.451 | 0.608 | 0.672 | 0.762 | 0.871 | 0.667 | 0.134 |
|  | Entire period of evaluation | 0.286 | 0.531 | 0.613 | 0.679 | 0.871 | 0.614 | 0.143 |
| GILÉ | Pre-MM | 0.321 | 0.392 | 0.471 | 0.570 | 0.662 | 0.474 | 0.110 |
|  | With-MM | 0.324 | 0.571 | 0.672 | 0.739 | 1.000 | 0.662 | 0.197 |
|  | Entire period of evaluation | 0.321 | 0.413 | 0.571 | 0.672 | 1.000 | 0.572 | 0.185 |
| ILE | Pre-MM | 0.333 | 0.395 | 0.494 | 0.637 | 0.714 | 0.517 | 0.133 |
|  | With-MM | 0.467 | 0.518 | 0.676 | 0.868 | 0.960 | 0.686 | 0.173 |
|  | Entire period of evaluation | 0.333 | 0.481 | 0.579 | 0.714 | 0.960 | 0.605 | 0.175 |
| INHASSUNGE | Pre-MM | 0.324 | 0.427 | 0.567 | 0.681 | 0.820 | 0.563 | 0.164 |
|  | With-MM | 0.476 | 0.792 | 0.845 | 0.987 | 1.000 | 0.840 | 0.169 |
|  | Entire period of evaluation | 0.324 | 0.560 | 0.736 | 0.845 | 1.000 | 0.707 | 0.216 |
| MAGANJA DA COSTA | Pre-MM | 0.394 | 0.458 | 0.507 | 0.566 | 0.833 | 0.528 | 0.120 |
|  | With-MM | 0.393 | 0.592 | 0.727 | 0.951 | 1.000 | 0.734 | 0.193 |
|  | Entire period of evaluation | 0.393 | 0.500 | 0.589 | 0.728 | 1.000 | 0.635 | 0.190 |
| MOCUBELA | Pre-MM | 0.202 | 0.479 | 0.669 | 0.859 | 0.972 | 0.651 | 0.254 |
|  | With-MM | 0.568 | 0.857 | 0.989 | 1.000 | 1.000 | 0.904 | 0.133 |
|  | Entire period of evaluation | 0.202 | 0.583 | 0.857 | 0.989 | 1.000 | 0.782 | 0.235 |
| MULEVALA | Pre-MM | 0.133 | 0.202 | 0.261 | 0.368 | 1.000 | 0.327 | 0.234 |
|  | With-MM | 0.357 | 0.583 | 0.750 | 1.000 | 1.000 | 0.763 | 0.244 |
|  | Entire period of evaluation | 0.133 | 0.273 | 0.500 | 1.000 | 1.000 | 0.553 | 0.323 |
| NAMACURRA | Pre-MM | 0.450 | 0.465 | 0.519 | 0.543 | 0.675 | 0.521 | 0.066 |
|  | With-MM | 0.408 | 0.626 | 0.669 | 0.818 | 0.912 | 0.701 | 0.153 |
|  | Entire period of evaluation | 0.408 | 0.515 | 0.555 | 0.675 | 0.912 | 0.615 | 0.149 |
| PEBANE | Pre-MM | 0.318 | 0.382 | 0.419 | 0.537 | 0.781 | 0.475 | 0.136 |
|  | With-MM | 0.429 | 0.590 | 0.734 | 0.812 | 0.972 | 0.695 | 0.155 |
|  | Entire period of evaluation | 0.318 | 0.429 | 0.550 | 0.742 | 0.972 | 0.589 | 0.182 |

**Table S13.** Proportion of infants with perinatal HIV exposure who received HIV DNA PCR testing by 9 months of age per month per district one year before (pre-MM) and one year after (with-MM) implementation of MM services.

| **District** | **Period** | **Min** | **Q1** | **Median** | **Q3** | **Max** | **Mean** | **SD** |
| --- | --- | --- | --- | --- | --- | --- | --- | --- |
| ALTO MOLÒCUÉ | Pre-MM | 0.508 | 0.713 | 0.793 | 0.910 | 0.941 | 0.793 | 0.129 |
|  | With-MM | 0.620 | 0.790 | 0.967 | 1.000 | 1.000 | 0.889 | 0.135 |
|  | Entire period of evaluation | 0.508 | 0.732 | 0.851 | 0.967 | 1.000 | 0.843 | 0.138 |
| GILÉ | Pre-MM | 0.464 | 0.740 | 0.877 | 0.911 | 0.983 | 0.808 | 0.159 |
|  | With-MM | 0.437 | 0.770 | 0.869 | 1.000 | 1.000 | 0.840 | 0.171 |
|  | Entire period of evaluation | 0.437 | 0.743 | 0.869 | 0.918 | 1.000 | 0.825 | 0.162 |
| ILE | Pre-MM | 0.479 | 0.595 | 0.795 | 0.936 | 1.000 | 0.769 | 0.199 |
|  | With-MM | 0.556 | 0.818 | 0.941 | 1.000 | 1.000 | 0.866 | 0.164 |
|  | Entire period of evaluation | 0.479 | 0.632 | 0.865 | 1.000 | 1.000 | 0.819 | 0.185 |
| INHASSUNGE | Pre-MM | 0.588 | 0.748 | 0.855 | 0.938 | 1.000 | 0.837 | 0.135 |
|  | With-MM | 0.635 | 0.939 | 1.000 | 1.000 | 1.000 | 0.922 | 0.127 |
|  | Entire period of evaluation | 0.588 | 0.761 | 0.939 | 1.000 | 1.000 | 0.881 | 0.135 |
| MAGANJA DA COSTA | Pre-MM | 0.705 | 0.802 | 0.923 | 1.000 | 1.000 | 0.889 | 0.112 |
|  | With-MM | 0.700 | 0.829 | 0.884 | 1.000 | 1.000 | 0.897 | 0.111 |
|  | Entire period of evaluation | 0.700 | 0.826 | 0.894 | 1.000 | 1.000 | 0.893 | 0.109 |
| MOCUBELA | Pre-MM | 0.797 | 1.000 | 1.000 | 1.000 | 1.000 | 0.969 | 0.072 |
|  | With-MM | 0.851 | 1.000 | 1.000 | 1.000 | 1.000 | 0.982 | 0.045 |
|  | Entire period of evaluation | 0.797 | 1.000 | 1.000 | 1.000 | 1.000 | 0.976 | 0.059 |
| MULEVALA | Pre-MM | 0.200 | 0.306 | 0.483 | 0.560 | 1.000 | 0.464 | 0.224 |
|  | With-MM | 0.583 | 0.786 | 1.000 | 1.000 | 1.000 | 0.888 | 0.147 |
|  | Entire period of evaluation | 0.200 | 0.500 | 0.700 | 1.000 | 1.000 | 0.684 | 0.284 |
| NAMACURRA | Pre-MM | 0.712 | 0.791 | 0.867 | 0.888 | 1.000 | 0.853 | 0.078 |
|  | With-MM | 0.647 | 0.787 | 0.898 | 0.954 | 1.000 | 0.867 | 0.112 |
|  | Entire period of evaluation | 0.647 | 0.787 | 0.883 | 0.914 | 1.000 | 0.861 | 0.096 |
| PEBANE | Pre-MM | 0.667 | 0.695 | 0.778 | 1.000 | 1.000 | 0.817 | 0.141 |
|  | With-MM | 0.643 | 0.827 | 0.932 | 1.000 | 1.000 | 0.902 | 0.113 |
|  | Entire period of evaluation | 0.643 | 0.772 | 0.837 | 1.000 | 1.000 | 0.861 | 0.132 |

**Table S14.** HIV DNA PCR positivity among infants with perinatal HIV exposure who received DNA PCR testing by 2 months of age per month per district one year before (pre-MM) and one year after (with-MM) implementation of MM services.

| **District** | **Period** | **Min** | **Q1** | **Median** | **Q3** | **Max** | **Mean** | **SD** |
| --- | --- | --- | --- | --- | --- | --- | --- | --- |
| ALTO MOLÒCUÉ | Pre-MM | 0.000 | 0.023 | 0.066 | 0.098 | 0.111 | 0.060 | 0.043 |
|  | With-MM | 0.000 | 0.000 | 0.022 | 0.079 | 0.104 | 0.038 | 0.038 |
|  | Entire period of evaluation | 0.000 | 0.018 | 0.032 | 0.088 | 0.111 | 0.048 | 0.041 |
| GILÉ | Pre-MM | 0.000 | 0.039 | 0.078 | 0.101 | 0.375 | 0.098 | 0.104 |
|  | With-MM | 0.000 | 0.000 | 0.065 | 0.139 | 0.182 | 0.075 | 0.068 |
|  | Entire period of evaluation | 0.000 | 0.031 | 0.065 | 0.105 | 0.375 | 0.086 | 0.087 |
| ILE | Pre-MM | 0.000 | 0.034 | 0.068 | 0.108 | 0.333 | 0.097 | 0.109 |
|  | With-MM | 0.000 | 0.000 | 0.044 | 0.107 | 0.250 | 0.068 | 0.072 |
|  | Entire period of evaluation | 0.000 | 0.000 | 0.049 | 0.107 | 0.333 | 0.082 | 0.091 |
| INHASSUNGE | Pre-MM | 0.000 | 0.015 | 0.062 | 0.108 | 0.167 | 0.068 | 0.059 |
|  | With-MM | 0.000 | 0.030 | 0.044 | 0.064 | 0.114 | 0.051 | 0.031 |
|  | Entire period of evaluation | 0.000 | 0.026 | 0.050 | 0.085 | 0.167 | 0.059 | 0.047 |
| MAGANJA DA COSTA | Pre-MM | 0.017 | 0.042 | 0.081 | 0.136 | 0.279 | 0.106 | 0.091 |
|  | With-MM | 0.000 | 0.029 | 0.045 | 0.068 | 0.114 | 0.051 | 0.032 |
|  | Entire period of evaluation | 0.000 | 0.029 | 0.060 | 0.091 | 0.279 | 0.077 | 0.071 |
| MOCUBELA | Pre-MM | 0.027 | 0.034 | 0.049 | 0.074 | 0.148 | 0.063 | 0.041 |
|  | With-MM | 0.000 | 0.022 | 0.032 | 0.047 | 0.083 | 0.034 | 0.022 |
|  | Entire period of evaluation | 0.000 | 0.027 | 0.042 | 0.056 | 0.148 | 0.048 | 0.035 |
| MULEVALA | Pre-MM | 0.000 | 0.000 | 0.000 | 0.000 | 0.143 | 0.012 | 0.041 |
|  | With-MM | 0.000 | 0.000 | 0.000 | 0.091 | 0.231 | 0.060 | 0.085 |
|  | Entire period of evaluation | 0.000 | 0.000 | 0.000 | 0.053 | 0.231 | 0.037 | 0.070 |
| NAMACURRA | Pre-MM | 0.020 | 0.029 | 0.040 | 0.058 | 0.121 | 0.050 | 0.030 |
|  | With-MM | 0.026 | 0.044 | 0.076 | 0.090 | 0.162 | 0.074 | 0.036 |
|  | Entire period of evaluation | 0.020 | 0.036 | 0.052 | 0.080 | 0.162 | 0.063 | 0.035 |
| PEBANE | Pre-MM | 0.000 | 0.046 | 0.071 | 0.090 | 0.138 | 0.067 | 0.037 |
|  | With-MM | 0.000 | 0.018 | 0.044 | 0.069 | 0.097 | 0.044 | 0.033 |
|  | Entire period of evaluation | 0.000 | 0.027 | 0.049 | 0.076 | 0.138 | 0.055 | 0.036 |

**Table S15.** HIV DNA PCR positivity among infants with perinatal HIV exposure who received DNA PCR testing by 9 months of age per month per district one year before (pre-MM) and one year after (with-MM) implementation of MM services.

| **District** | **Period** | **Min** | **Q1** | **Median** | **Q3** | **Max** | **Mean** | **SD** |
| --- | --- | --- | --- | --- | --- | --- | --- | --- |
| ALTO MOLÒCUÉ | Pre-MM | 0.019 | 0.052 | 0.066 | 0.143 | 0.200 | 0.097 | 0.060 |
|  | With-MM | 0.000 | 0.016 | 0.041 | 0.100 | 0.159 | 0.054 | 0.050 |
|  | Entire period of evaluation | 0.000 | 0.020 | 0.062 | 0.109 | 0.200 | 0.074 | 0.058 |
| GILÉ | Pre-MM | 0.000 | 0.037 | 0.104 | 0.128 | 0.270 | 0.102 | 0.079 |
|  | With-MM | 0.000 | 0.055 | 0.081 | 0.129 | 0.157 | 0.083 | 0.055 |
|  | Entire period of evaluation | 0.000 | 0.038 | 0.097 | 0.129 | 0.270 | 0.092 | 0.067 |
| ILE | Pre-MM | 0.000 | 0.049 | 0.141 | 0.195 | 0.292 | 0.138 | 0.096 |
|  | With-MM | 0.000 | 0.031 | 0.037 | 0.125 | 0.231 | 0.076 | 0.072 |
|  | Entire period of evaluation | 0.000 | 0.031 | 0.094 | 0.162 | 0.292 | 0.106 | 0.088 |
| INHASSUNGE | Pre-MM | 0.000 | 0.039 | 0.098 | 0.157 | 0.308 | 0.116 | 0.099 |
|  | With-MM | 0.000 | 0.050 | 0.078 | 0.103 | 0.135 | 0.072 | 0.039 |
|  | Entire period of evaluation | 0.000 | 0.046 | 0.085 | 0.116 | 0.308 | 0.093 | 0.076 |
| MAGANJA DA COSTA | Pre-MM | 0.059 | 0.088 | 0.140 | 0.194 | 0.286 | 0.153 | 0.084 |
|  | With-MM | 0.009 | 0.060 | 0.076 | 0.095 | 0.157 | 0.076 | 0.041 |
|  | Entire period of evaluation | 0.009 | 0.063 | 0.095 | 0.157 | 0.286 | 0.113 | 0.075 |
| MOCUBELA | Pre-MM | 0.012 | 0.069 | 0.084 | 0.127 | 0.297 | 0.101 | 0.071 |
|  | With-MM | 0.000 | 0.019 | 0.048 | 0.064 | 0.091 | 0.044 | 0.030 |
|  | Entire period of evaluation | 0.000 | 0.029 | 0.064 | 0.084 | 0.297 | 0.071 | 0.060 |
| MULEVALA | Pre-MM | 0.000 | 0.000 | 0.000 | 0.000 | 0.125 | 0.010 | 0.036 |
|  | With-MM | 0.000 | 0.000 | 0.048 | 0.118 | 0.214 | 0.062 | 0.075 |
|  | Entire period of evaluation | 0.000 | 0.000 | 0.000 | 0.053 | 0.214 | 0.037 | 0.064 |
| NAMACURRA | Pre-MM | 0.028 | 0.071 | 0.100 | 0.126 | 0.270 | 0.113 | 0.068 |
|  | With-MM | 0.060 | 0.063 | 0.098 | 0.098 | 0.168 | 0.092 | 0.030 |
|  | Entire period of evaluation | 0.028 | 0.063 | 0.098 | 0.116 | 0.270 | 0.102 | 0.052 |
| PEBANE | Pre-MM | 0.000 | 0.082 | 0.134 | 0.142 | 0.280 | 0.120 | 0.069 |
|  | With-MM | 0.000 | 0.053 | 0.063 | 0.083 | 0.203 | 0.069 | 0.052 |
|  | Entire period of evaluation | 0.000 | 0.058 | 0.083 | 0.139 | 0.280 | 0.094 | 0.065 |

**Supplemental Figures**





**Figure S1**. One-month retention rate for pregnant and postpartum women living with HIV (PPWH). The vertical dashed line represents the start of Mentor Mother (MM) implementation, with the 12 months prior to MM implementation to the left and 12 months with MM to the right. The red line represents what happened in the pre-MM period and projects what would have happened if MM had not been implemented. The blue line represents what was observed after MM implementation. The shaded areas represent the 95% confidence interval of the fitted lines. The dots show the raw data used for regression analysis.





**Figure S2**. Three-month retention rate for pregnant and postpartum women living with HIV (PPWH). The vertical dashed line represents the start of Mentor Mother (MM) implementation, with the 12 months prior to MM implementation to the left and 12 months with MM to the right. The red line represents what happened in the pre-MM period and projects what would have happened if MM had not been implemented. The blue line represents what was observed after MM implementation. The shaded areas represent the 95% confidence interval of the fitted lines. The dots show the raw data used for regression analysis.





**Figure S3**. Six-month retention rate for pregnant and postpartum women living with HIV (PPWH). The vertical dashed line represents the start of Mentor Mother (MM) implementation, with the 12 months prior to MM implementation to the left and 12 months with MM to the right. The red line represents what happened in the pre-MM period and projects what would have happened if MM had not been implemented. The blue line represents what was observed after MM implementation. The shaded areas represent the 95% confidence interval of the fitted lines. The dots show the raw data used for regression analysis.





**Figure S4**. Twelve-month retention rate for pregnant and postpartum women living with HIV (PPWH). The vertical dashed line represents the start of Mentor Mother (MM) implementation, with the 12 months prior to MM implementation to the left and 12 months with MM to the right. The red line represents what happened in the pre-MM period and projects what would have happened if MM had not been implemented. The blue line represents what was observed after MM implementation. The shaded areas represent the 95% confidence interval of the fitted lines. The dots show the raw data used for regression analysis.





**Figure S5**. Viral suppression rate for pregnant and postpartum women living with HIV (PPWH). The vertical dashed line represents the start of Mentor Mother (MM) implementation, with the 12 months prior to MM implementation to the left and 12 months with MM to the right. The red line represents what happened in the pre-MM period and projects what would have happened if MM had not been implemented. The blue line represents what was observed after MM implementation. The shaded areas represent the 95% confidence interval of the fitted lines. The dots show the raw data used for regression analysis.





**Figure S6**. HIV DNA PCR uptake among infants with perinatal HIV exposure by 2 months of age. The vertical dashed line represents the start of Mentor Mother (MM) implementation, with the 12 months prior to MM implementation to the left and 12 months with MM to the right. The red line represents what happened pre-MM and projects what would have happened if MM had not been implemented. The blue line represents what was observed after MM implementation. The shaded areas represent the 95% confidence interval of the fitted lines. The dots show the raw data used for regression analysis.





**Figure S7**. HIV DNA PCR uptake among infants with perinatal HIV exposure by 9 months of age. The vertical dashed line represents the start of Mentor Mother (MM) implementation, with the 12 months prior to MM implementation to the left and 12 months with MM to the right. The red line represents what happened in pre-MM and projects what would have happened if MM had not been implemented. The blue line represents what was observed after MM implementation. The shaded areas represent the 95% confidence interval of the fitted lines. The dots show the raw data used for regression analysis.





**Figure S8**. HIV DNA PCR positivity among infants with perinatal HIV exposure tested from 0-2 months of age. The vertical dashed line represents the start of Mentor Mother (MM) implementation, with the 12 months prior to MM implementation to the left and 12 months with MM to the right. The red line represents what happened pre-MM and projects what would have happened if MM had not been implemented. The blue line represents what was observed after MM implementation. The shaded areas represent the 95% confidence interval of the fitted lines. The dots show the raw data used for regression analysis.





**Figure S9**. HIV DNA PCR positivity among infants with perinatal HIV exposure tested from 0-9 months of age. The vertical dashed line represents the start of Mentor Mother (MM) implementation, with the 12 months prior to MM implementation to the left and 12 months with MM to the right. The red line represents what happened the pre-MM and projects what would have happened if MM had not been implemented. The blue line represents what was observed after MM implementation. The shaded areas represent the 95% confidence interval of the fitted lines. The dots show the raw data used for regression analysis.
